# The *Arabidopsis* RCC1 Family Protein TCF1 Regulates Freezing Tolerance and Cold Acclimation through Modulating Lignin Biosynthesis

**DOI:** 10.1371/journal.pgen.1005471

**Published:** 2015-09-22

**Authors:** Hongtao Ji, Youning Wang, Catherine Cloix, Kexue Li, Gareth I. Jenkins, Shuangfeng Wang, Zhonglin Shang, Yiting Shi, Shuhua Yang, Xia Li

**Affiliations:** 1 State Key Laboratory of Plant Cell and Chromosome Engineering, Center for Agricultural Research Resources, Institute of Genetics and Developmental Biology, Chinese Academy of Sciences, Shijiazhuang, Hebei, China; 2 State Key Laboratory of Agricultural Microbiology, College of Plant Science and Technology, Huazhong Agricultural University, Wuhan, China; 3 Institute of Molecular Cell and Systems Biology, College of Medical, Veterinary and Life Sciences, Bower Building, University of Glasgow, Glasgow, United Kingdom; 4 College of Life Science, Hebei Normal University, Shijiazhuang, China; 5 The State Key Laboratory of Plant Physiology and Biochemistry, College of Biological Sciences, China Agricultural University, Beijing, China; The University of North Carolina at Chapel Hill, UNITED STATES

## Abstract

Cell water permeability and cell wall properties are critical to survival of plant cells during freezing, however the underlying molecular mechanisms remain elusive. Here, we report that a specifically cold-induced nuclear protein, Tolerant to Chilling and Freezing 1 (TCF1), interacts with histones H3 and H4 and associates with chromatin containing a target gene, *BLUE-COPPER-BINDING PROTEIN* (*BCB*), encoding a glycosylphosphatidylinositol-anchored protein that regulates lignin biosynthesis. Loss of *TCF1* function leads to reduced *BCB* transcription through affecting H3K4me2 and H3K27me3 levels within the *BCB* gene, resulting in reduced lignin content and enhanced freezing tolerance. Furthermore, plants with knocked-down *BCB* expression (*amiRNA-BCB*) under cold acclimation had reduced lignin accumulation and increased freezing tolerance. The *pal1pal2* double mutant (lignin content reduced by 30% compared with WT) also showed the freezing tolerant phenotype, and *TCF1* and *BCB* act upstream of *PALs* to regulate lignin content. In addition, *TCF1* acts independently of the *CBF* (C-repeat binding factor) pathway. Our findings delineate a novel molecular pathway linking the TCF1-mediated cold-specific transcriptional program to lignin biosynthesis, thus achieving cell wall remodeling with increased freezing tolerance.

## Author Summary

Cold acclimation is a well-known adaptive process through which plants can dramatically increase their tolerance to freezing temperature. Modifications of cell wall have been recognized as a key characteristic during plant acclimation to low temperature. However, the molecular mechanism responsible for such cellular adaptation still remains a mystery. Here, we report an unexpected regulatory role of *TCF1* on lignin content during cold acclimation in *Arabidopsis*. *TCF1* is specifically induced by cold and is required for chromatin based gene regulation of cold responsive genes such as *BCB* (a GAP) that regulates lignin genes. Further evidence shows that reduction in lignin dramatically increases plant freezing tolerance, while lignin maintenance required for cold acclimation is regulated by TCF-mediated signaling. Thus, our study has revealed, for the first time, lignin remodeling as a key function of cold acclimation and freezing tolerance. The findings provide the first direct molecular evidence that freezing tolerance is directly related to cell wall properties during cold acclimation and extra/intercellular freezing upon and freezing/thawing process.

## Introduction

Freezing temperature is an important environmental factor that determines the natural geographical distribution of plants and limits crop productivity [1]. Sudden exposure to freezing temperature causes intracellular freezing, membrane damage and cell death [1–3]. To better survive freezing low temperature, plants have evolved coping mechanisms through initiating cold acclimation when the temperature gradually drops lower in autumn in nature. Many signal transduction cascades are involved in this physiological adaptation process. In *Arabidopsis*, expression profiling of cold-treated plants revealed that up to 20% of genes in the genome are regulated by cold. Characterization of a group of cold-regulated (*COR*) genes which are highly induced by cold stress using forward and reverse genetics has led to identification of a key CBF (C-repeat binding factor, also known as dehydration-responsive element-binding protein 1 or DREB1) signaling pathway. CBF transcription factors (CBF1, CBF2, CBF3) can activate expression of the *COR* genes by binding to *cis*-elements in their promoters and induce cold acclimation and freezing tolerance [4–6]. Several regulators of *CBF* genes have been identified, such as Inducer of CBF expression 1 (ICE1), calmodulin binding transcription activator 3 (CAMTA3), MYB15 and Ethylene Insensitive 3 (EIN3) [7–10]. Most recently, it has been shown that OPEN STOMATA 1 (OST1), a central component in ABA signaling pathway, plays a crucial role in plant response to cold. OST1 is induced by cold and cold-activated OST1 can interact and phosphorylate ICE1 to enhance the stability of ICE1, resulting in increased plant tolerance to freezing [11]. However, multiple studies have reported that the CBF signaling pathway is not the sole mechanism modulating plant cold acclimation and cold tolerance, because only 12% of the cold responsive genes are regulated by CBF transcription factors [12]. The prominent example is HOS15, which regulates freezing tolerance through modification of histone acetylation [13,14]. In addition, SFR2 was found to modulate freezing tolerance through lipid remodeling of the outer chloroplast membrane [15]. Most recently, it was found that AtHAP5A modulates freezing stress resistance in *Arabidopsis* independent of the CBF pathway [16]. Through these signaling pathways, a wide variety of antifreeze/stress-related proteins and compounds are accumulated to minimize intracellular ice formation, to increase tolerance to dehydration caused by water outflow, and to maintain cell membrane stability and integrity that is considered central to the ability of plants to survive freezing [2,15,17–19].

The plant cell wall is the extracellular matrix consisting of cellulose, hemicellulose and lignin. It plays essential roles in plant growth and adaptive responses to adverse environmental conditions [20–22]. The cell wall integrity (CWI) and structures are dynamically regulated during plant development and are capable of being remodeled in response to various environmental stresses [23–26]. Fine-tuning regulation of the proportions and the amounts of each matrix component within the cell wall determines its nature and functions. Remarkably, deposition of lignin, phenylpropanoid polymer, which is highly hydrophobic in the cell wall, determines cell wall stiffness and permeability to water [27–29]. In yeast, the CWI signaling pathway plays a vital role in adjusting the cell wall thickness and composition to environmental cues, in particular freezing temperature and osmotic stress [30,31]. In plants, similar processes are employed for controlling cell wall integrity and performance during development, drought and defense [32], but the precise mechanisms remain unclear.

Previous studies have shown that the expression of genes related to cell wall biosynthesis and remodeling is dramatically altered under cold treatment [33]. Using cryo-scanning electron microscopy (cryo-SEM), several studies have revealed that both cell membrane and cell wall properties play equally important roles in cold acclimation and freezing tolerance [18]. Most strikingly, cell wall thickness and rigidity have been linked to dynamic water heterogeneities during cold acclimation and extra-/inter- or intracellular freezing upon freezing/thawing process. Variations in cell wall rigidity and composition in different types of plant tissues and cells (e.g. xylem, phloem, living fibers and mesophyll cells) showed altered intracellular freezing, tension-induced cavitation and cell viability during freezing/thawing [34–36]. Thus, resistance to freezing temperatures is dependent on the capacity for water outflow from the cells during cold acclimation and freezing and water reabsorption during thawing, on the capacity to accommodate growth of ice crystals in extra-/intercellular spaces, and on the ability of cell wall elasticity to respond to cellular shrinkage.

Investigation of the roles of the cell wall in cold acclimation and freezing/thawing has been limited to correlation of cellular changes in various plant species or different types of tissues and cells using cryo-SEM; mechanistic analysis of the regulatory genes and signaling cascades underlying cell wall mediated water movement and freezing tolerance is lacking. Lignin is a major component of the plant secondary cell wall, and the amounts of lignin are altered after cold treatment in various species [37,38]. In the past decades, some genes that regulate lignin biosynthesis have been identified [39–43]. Among them, *Phenylalanine ammonia-lyase 1–4* (PAL; EC 4.3.1.5) encoding the enzymes that catalyze the first step in the phenylpropanoid pathway regulate biosynthesis of lignin and secondary metablites (e.g. flavonoids and salicylic acid) in *Arabidopsis thaliana* [39,44–48]. *Arabidopsis thaliana* blue copper binding gene (*BCB*) is another positive regulator of lignin synthesis, and *AtBCB* overexpression substantially increases lignin content in *Arabidopsis* roots [49]. It has been shown that *PAL1-PAL4* and *BCB* genes are responsive to a variety of environmental stimuli, including pathogen infection, wounding, nutrient depletion, UV irradiation, and extreme temperature, etc. [49,50], suggesting their roles in plant stress resistance. However, it remains unknown how these genes mediate plant responses to biotic and abiotic stresses.

Here we report that *T* *olerant to* *C* *hilling and* *F* *reezing 1* (*TCF1*), a gene encoding a RCC1 family protein, is required for chromatin based gene regulation of cold responsive genes in a CBF-independent pathway. Importantly, we reveal that lignin content in leaves is directly related to freezing tolerance, and that TCF1 plays a critical role in the adjustment of lignin accumulation through modulation of expression of *BCB* and downstream effectors *PAL1/3/4* genes during cold acclimation and freezing tolerance in *Arabidopsis*.

## Results

### Identification and Characterization of the *TCF1* Gene

**Fig 1:**
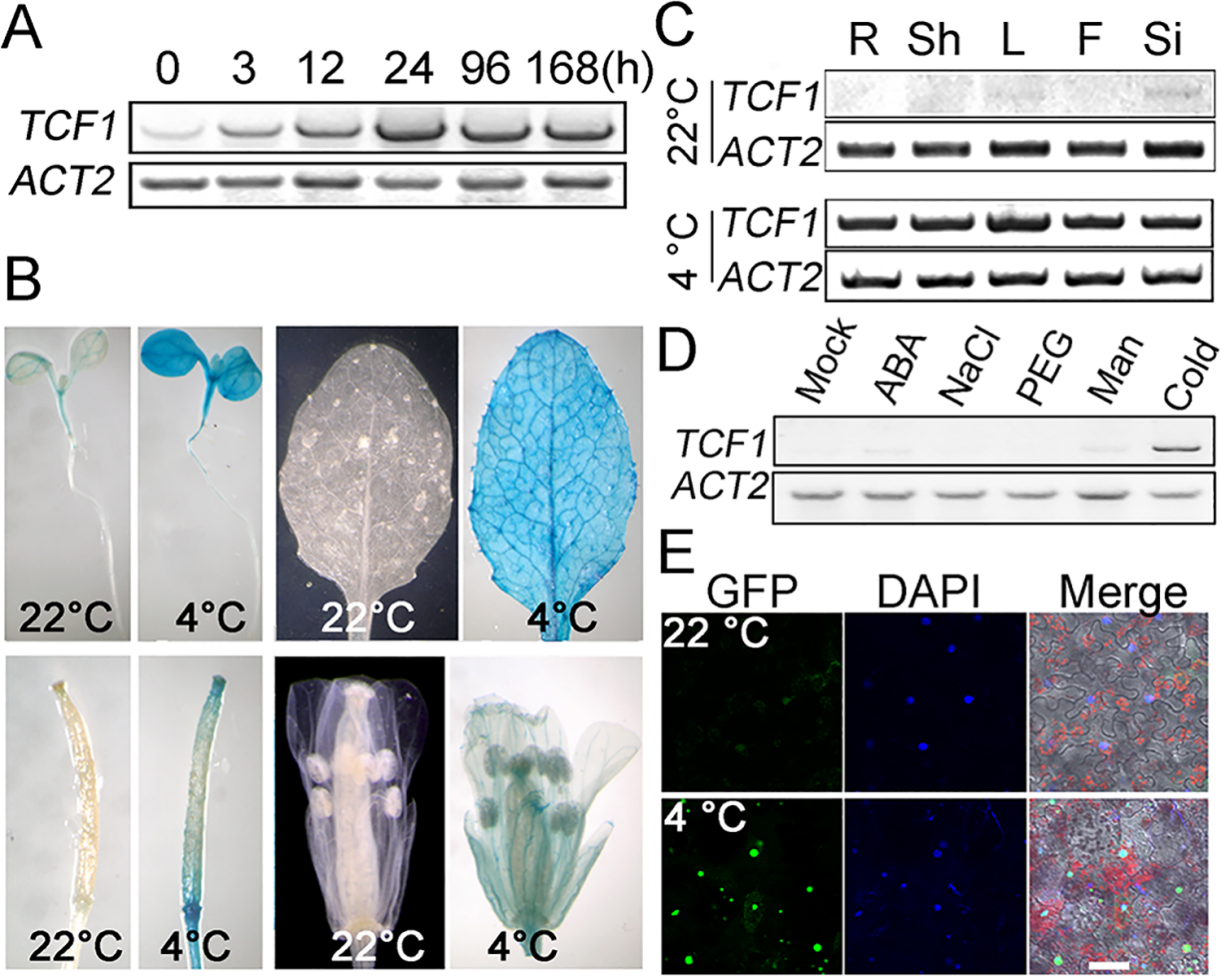
Analysis of *TCF1* expression and nuclear localization of the TCF1 protein. **(A)** Three-week-old Col-0 (WT) plants were subjected to low temperature (4°C) and the samples were harvested at the indicated time points for semi-quantitative RT-PCR analysis of *TCF1* transcripts. *ACT2* (*At3g18780*) was used as a loading control. **(B)** GUS staining of transgenic plants expressing *TCF1pro*::*GUS* under normal temperature or treated at 4°C for 7 days. **(C)** Semi-quantitative RT-PCR for *TCF1* in different tissues with or without cold treatments for 7 days. R, root; Sh, shoot; L, leaves; F, flowers; Si, siliques. **(D)** Semi-quantitative RT-PCR analysis for *TCF1* of three-week-old Col-0 plants treated with 100 μM ABA, 400 mM mannitol, 20% PEG6000, 300 mM NaCl for 3 h and 4°C Cold treatment for 24 h. **(E)** Localization of fluorescence in *tcf1-1* plants expressing a *TCF1pro*::*GFP-TCF1* fusion at 22°C (*Upper*) and 4°C for 7 days (*Bottom*). GFP: GFP-TCF1 fusion protein (*tcf1-1TCF1-3*), DAPI: DAPI staining, Merge: Merger of GFP and DAPI channels (Scale bars, 20 μm).

To identify the genetic loci that regulate specifically plant cold acclimation and freezing tolerance through chromatin condensation and remodeling, we examined cold responses of the genes encoding Regulator of Chromatin Condensation 1 (RCC1) family proteins from AtGenExpress Data [51]. The gene *At3g55580* which is specifically responsive to cold was identified (S1A Fig), and designated *T* *olerant to* *C* *hilling and* *F* *reezing1* (*TCF1*) based on the phenotypes of its mutant. RT-PCR analysis and GUS assay of *TCF1pro*::*GUS* lines validated induction of *TCF1* expression in response to cold but not to osmotic stress or ABA (Fig 1A–1D).

The *TCF1* gene encodes a protein containing six predicted tandem RCC1 repeats that shows similarity to RCC1 in yeast and human [52,53] (S1B and S1C Fig). To determine whether TCF1 is localized in the nucleus like RCC1 [54,55], we made translational fusions with GFP and expressed them in *tcf1-1* plants using the native promoters. Examination of independent transgenic lines revealed that GFP-TCF1 fluorescence was present in the nucleus (Fig 1E), and the level of the fusion protein GFP-TCF1 was also induced by cold (Fig 1E).

### TCF1 Interacts with Histones and Has Negligible GEF Activity *in Vitro*

**Fig 2:**
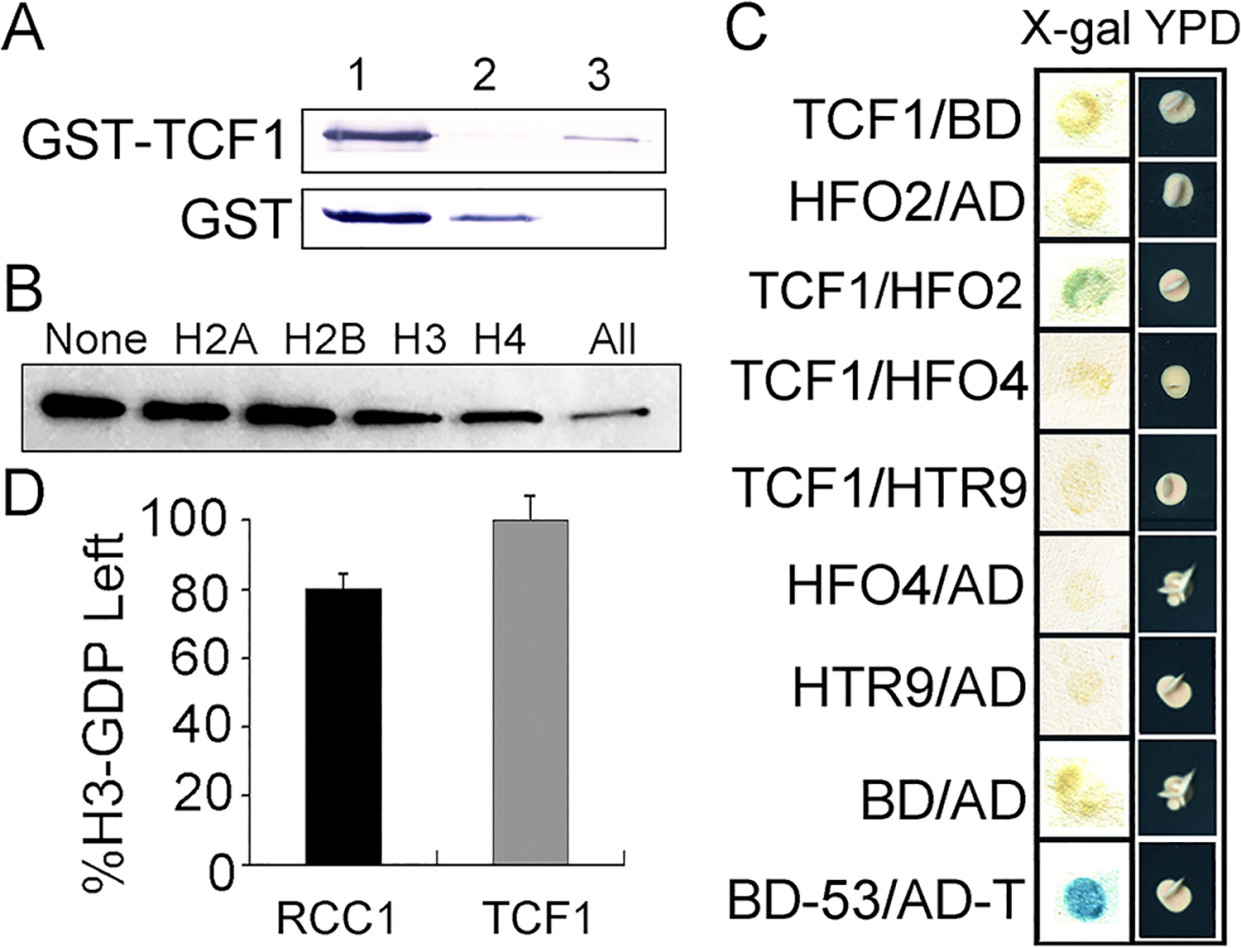
GEF activity assay of TCF1 and analysis of TCF1-histone interactions. **(A)** Binding of *E*. *coli*-expressed GST-TCF1 to a calf thymus histone-agarose column. Western blots with anti-GST antibody showing GST-TCF1 (77 kDa) or control GST (26 kDa). Lane 1, protein applied to the columns; lane 2, unbound material that flowed through; lane 3, protein bound after a 5 min incubation, eluted with 0.3 M NaCl. **(B)** 0.3 μg of *in vitro* translated Myc-TCF1 was incubated with 20 μg of the appropriate histone (20-fold excess) for 30 min at room temperature. The samples were then incubated with histone-agarose overnight at 4°C. Myc-TCF1 binding to histone-agarose was analyzed by Western blot. The different histones used as competitors are indicated at the top of each lane. ‘All’ indicates the mixture of histones and ‘None’ indicates the control without competitors. **(C)** Yeast co-transformant strains carrying both *TCF1* and vector control (TCF1/BD), *histone H3/H4* and vector control (HFO2/AD, HFO4/AD and HTR9/AD), *TCF1* and *H3*/*H4* (TCF1/HFO2,TCF1/HFO4 and TCF1/HTR9), negative control (BD/AD) and positive control (BD-53/AD-T) were streaked onto selective media. Activation of the *lacZ* reporter gene is indicated by the formation of blue or blue-green colonies on plates containing X-Gal (*left*), the growth state of yeast co-transformant strains in YPD medium is shown on the right (*right*). **(D)** Ran-GEF activity of *E*. *coli*-expressed GST-RCC1 and GST-TCF1 is shown as the percentage of [^3^H]GDP remaining at the end of the GEF assay.

RCC1 is a guanine nucleotide exchange factor (GEF) for the small GTP-binding protein Ran. RCC1 is constitutively localized in the nucleus, binds to chromatin, and generates a Ran-GTP/Ran-GDP gradient across the nuclear envelope that is required both to drive nucleo-cytoplasmic transport and to regulate processes associated with progression of the cell cycle and mitosis [54,55]. We then ask whether TCF1 has the similar roles of RCC1. To address whether TCF1 interacts physically with chromatin, an *in vitro* assay was performed. The fusion protein GST-TCF1 expressed in *E*. *coli* bound strongly to a histone agarose column (Fig 2A). To further investigate which histone TCF1 interacts preferentially with, *in vitro* translated Myc-tagged TCF1 was pre-incubated with each kind of purified core histone in 20-fold excess followed by incubation with histone-agarose. As shown in Fig 2B, H3 and H4 were the histones that can compete effectively to diminish the binding of Myc-TCF1 to histone-agarose, suggesting high affinity binding between TCF1 and histones H3 and H4. Further yeast-two-hybrid results confirmed that TCF1 can indeed interact with the specific histone H4 (HFO2, at5g59690), but not other tested histone H3 and H4s (HTR9, At5g10400; HFO4, At1g07820) (Fig 2C). To exam whether TCF1 has GEF activity, an *in vitro* assay was performed. The results showed that TCF1 exhibited less than 1% of the GEF activity with human Ran as substrate of that measured for RCC1 (Fig 2D). Also expression of TCF1 did not complement the phenotype of the yeast *prp20* mutant lacking yeast RCC1 (S2 Fig). The results indicate that TCF1 is not the ortholog of RCC1 in *Arabidopsis*, but it does associate with chromatin via its interaction with histones.

### *TCF1* Mutant Plants Are Tolerant to Freezing

**Fig 3:**
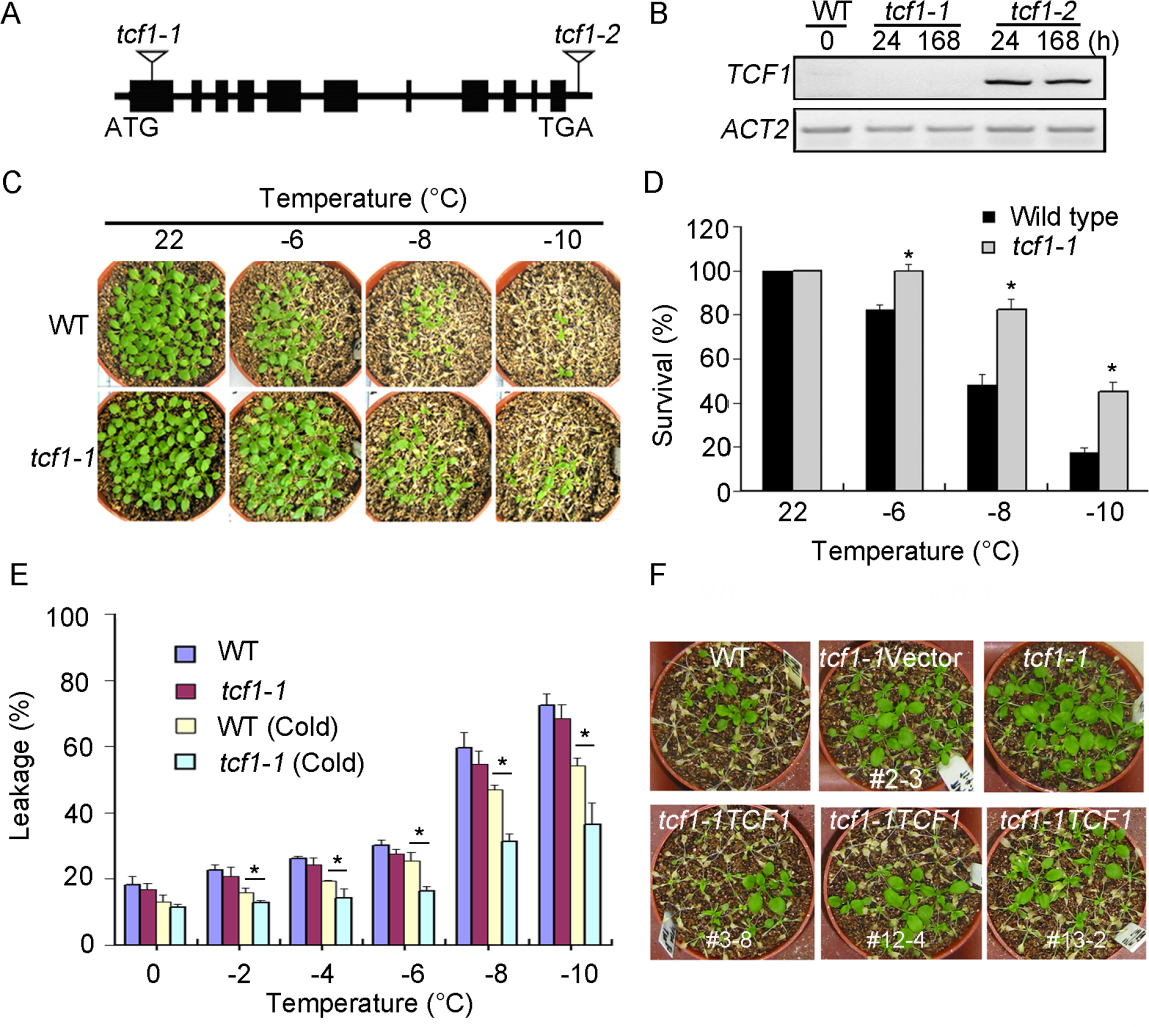
*tcf1-1* plants are tolerant to freezing treatment. **(A)** Schematic presentation of the *TCF1* gene structure and T-DNA insertions in the *TCF1* gene (arrowheads). The closed rectangles represent exons and lines between the exons denote introns. **(B)** *tcf1-1* is a null mutation of *TCF1*. The levels of *TCF1* transcripts were determined by RT-PCR using 3-week-old *tcf1-1* seedlings subjected to low temperature (4°C) for the indicated time periods (h); the *ACT2* gene was used as loading control. **(C)** Tolerance of 3-week-old *tcf1-1* and wild-type (WT) plants at the indicated temperatures below freezing under long-day photoperiod with cold acclimation for 7 days. The pictures were taken 7 days after treatments. **(D)** Quantification of survival rate of the treated plants in **(C)**, (*, *P* < 0.05, *t*-test). **(E)** Leakage of electrolytes in *tcf1-1* and WT plants treated at indicated temperatures below freezing. WT (cold) and *tcf1-1* (cold): 3-week-old plants were cold-acclimated (4°C for 7 day), WT and *tcf1-1*: both plants were grown under normal conditions. Error bars are standard deviation (*n* = 8), (*, *P* < 0.05, *t*-test). **(F)** Tolerance of freezing treatments (-8°C for 2 h) of control and transgenic plants, which were cold-acclimated at 4°C for 7 days before the treatment. The plants included WT, *tcf1-1*, representative homozygous lines of *tcf1-1* transformed with an empty vector pEZR(K)LC (*tcf1-1Vector*-2 (#2–3)) or *TCF1* gene (*tcf1-1TCF1-3* (#3–8), *tcf1-1TCF1-12* (#12–4) and *tcf1-1TCF1-13* (#13–2)).

To verify the function of TCF1 in cold and/or freezing response, *tcf1-1* and *tcf1-2* were analyzed (Fig 3A and 3B). *tcf1-1* with T-DNA insertion at the first exon of *TCF1* showed no *TCF1* transcript in response to cold treatment (Fig 3B), but *tcf1-2* having a T-DNA insertion in the 3’-UTR region of *TCF1* (Fig 3A) exhibited similar *TCF1* expression to that of the wild-type under cold treatment (Fig 3B). Thus, the phenotypic analysis of *tcf1-1* was shown thereafter. All of the F1 plants from *tcf1-1*×wild-type cross were resistant on MS medium containing 5 mg/L bialaphos. The F2 progeny of the selfed F1 plants segregated in a 3: 1 ratio (From 3130 plants, 2322 conferring resistant to bialaphos compared with 808 plants showing sensitive phenotype, x^2^ = 1.11 < 3.841; x^2^ test with one degree of freedom). Analysis of the bialaphos resistance revealed the presence of a single functional T-DNA that is inserted in the genome of the *tcf1-1* mutant. The results indicated that the *tcf1-1* mutation is recessive in a single nuclear gene.

To evaluate the effect of the *tcf1-1* mutation on freezing tolerance, we performed the whole-plant freezing test. Without cold acclimation, *tcf1-1* shows a slightly higher survival rate than wild-type, but there was no significant difference between wild-type and *tcf1-1* (S3A Fig). When the plants were acclimated at 4°C for 7 days, 45.2% of the *tcf1-1* plants survived freezing temperature as low as -10°C, but only 17.4% of the wild-type plants survived the treatment (Fig 3C and 3D). The electrolyte leakage assay confirmed the freezing tolerance of *tcf1-1* plants under cold acclimation (Fig 3E). To verify the role of *TCF1* in cold acclimation, we generated *TCF1* RNA interference (*TCF1-RNAi*) lines and two *TCF1-RNAi* lines (*TCF1-RNAi-2* and *TCF1-RNAi-6*) with reduced expression of *TCF1* were used in freezing response assay (S3B Fig). Without cold acclimation, both *TCF1-RNAi lines* showed a similar survival rate to the wild-type (S3C Fig). When the plants were acclimated at 4°C for 7 days, these *TCF1-RNAi* lines also displayed significantly higher survival rates than the wild-type plants at -10°C (S3D and S3E Fig). The percentages of electrolyte leakage of *TCF1-RNAi* lines were also decreased under cold acclimation (S3F Fig), suggesting a role of *TCF1* during cold acclimation and freezing tolerance in *Arabidopsis*.

To further determine that freezing tolerance of *tcf1-1* was due to loss of function in *TCF1*, we expressed the GFP-CDS of *TCF1* under the control of its native promoter in the *tcf1-1* background (Fig 1E). Three independent transgenic lines (*tcf1-1TCF1-3*, *tcf1-1TCF1-12* and *tcf1-1TCF1-13*) with increased levels of *TCF1* showed a cold-sensitive phenotype compared with *tcf1-1* mutant and had a similar response to the wild-type under freezing treatment with cold acclimation (Figs 3F and S4), thereby confirming that expression of *TCF1* complemented the freezing tolerance phenotype of the mutant.

### *TCF1* Regulates Freezing Tolerance through a CBF Independent Pathway

**Fig 4:**
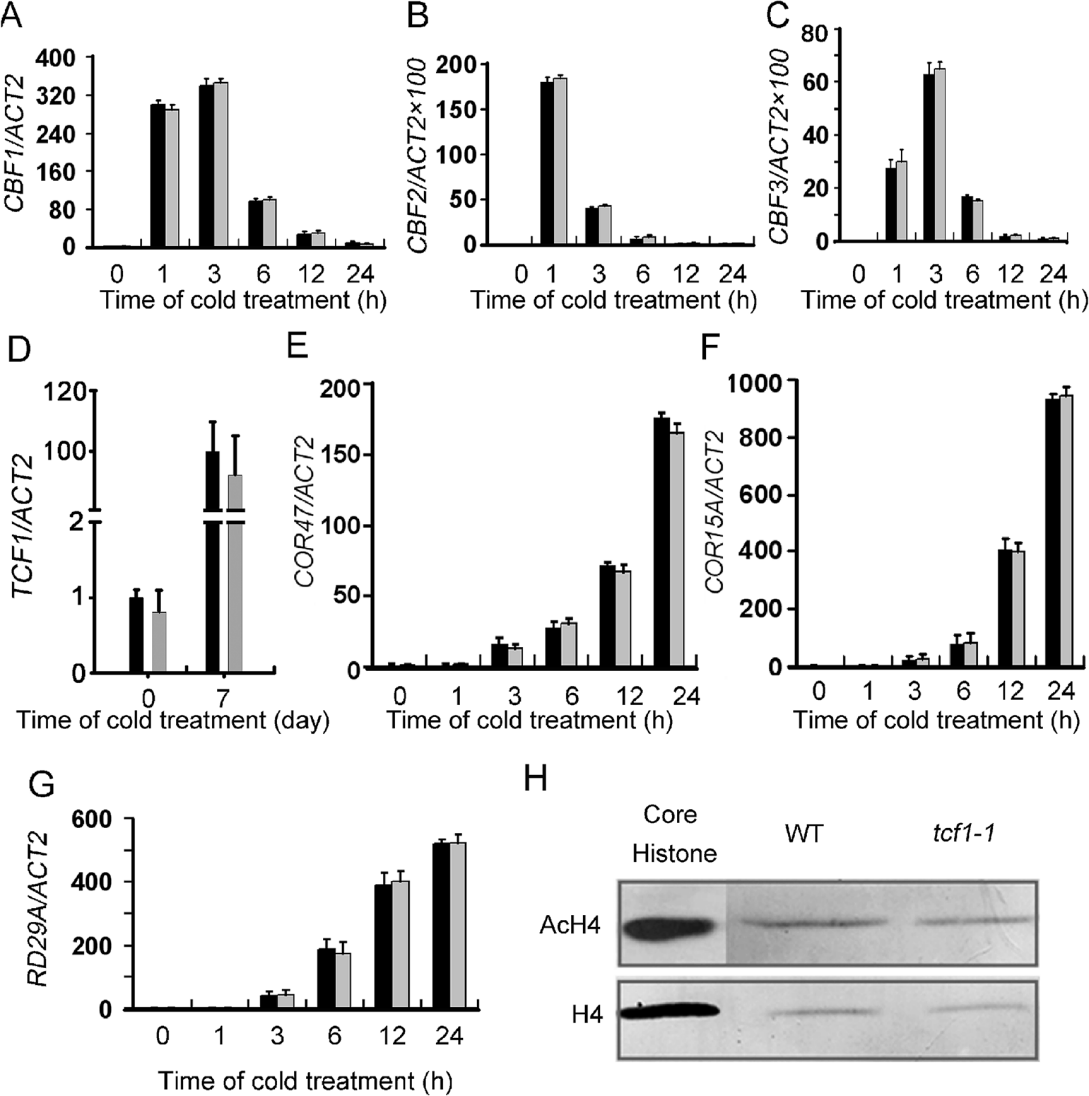
Gene expression analyses of the CBFs and the CBF regulon in the *tcf1-1* and wild-type plants. **(A)** to **(C)** The levels of *CBF1*, *CBF2* and CBF3 transcripts, respectively, in *tcf1-1* and wild-type plants. The plants were treated with cold (4°C) at the indicated time points. **(D)** The level of *TCF1* transcripts in the loss-of-function *cbf2* mutant plants grown under normal conditions or given a 4°C treatment for 7 day. Transcript levels of the *ACT2* gene were used as a loading control. **(E)** to **(G)** Transcript levels of *COR47* **(E)**, *COR15A* **(F)** and *RD29A* **(G)** in *tcf1-1* and wild-type plants. The plants were treated with cold (4°C) for the indicated times. **(H)** Western blot analysis with anti-acetylated H4 antibody (*Top*) revealed that *tcf1-1* and WT plants have similar levels of acetylated H4. Histone H4 antibody for core histone H4 was used as the immunoblot control (*Bottom*).

Because TCF1 associates with chromatin, we questioned whether TCF1 regulates freezing tolerance by modulating expression of *CBF1-3* and the targeted genes. However, the expression levels and patterns of *CBF1*-*3* under cold treatment in *tcf1-1* did not show any significant difference from that in the wild-type (Fig 4A–4C and S5A). No significant changes in transcript levels of *TCF1* in *cbf2* (S5B Fig) were detected under cold (Fig 4D). In addition, the expression of the *CBF* regulon genes such as *COR15A* and *COR47* was also not changed in *tcf1-1* under cold (Fig 4E and 4F). Notably, *TCF1* was not in the list of the *CBF1*, *CBF2* and *CBF3* coregulated genes in the previous study [12]. These results indicate that *TCF1* may influence cold/freezing tolerance through a mechanism different from the CBF-COR cascade.

Because *TCF1* reached the highest level of expression and loss of function in *TCF1* enhanced freezing tolerance with a 7-day cold acclimation, the expression profiles of *tcf1-1* and the wild-type grown under normal condition with a 7-day cold acclimation and without cold acclimation were compared by the GeneChip Array. Under normal conditions, only 20 genes up-regulated or down-regulated (> 1.5 fold) in *tcf1-1* were identified and they are annotated to encode proteins with diverse cellular functions (S1 Table). Five genes that should be repressed during cold exposure were activated in *tcf1-1*, and 12 down-regulated genes are involved in diverse stress response processes. After cold acclimation, expression of 36 genes was varied (13 genes up-regulated and 23 down-regulated) in *tcf1-1* (S2 Table). Among them, nineteen genes were cold responsive and are involved in different cell functions, but none of them appears to involve the CBF regulon. The highest and lowest two expression genes in microarray data under cold acclimation were validated by qRT-PCR (S6 Fig). The data supports that *TCF1* functions in a novel pathway independent of the CBF-COR cascade.

### TCF1 Does Not Affect Histone H4 Acetylation

*HOS15* has been reported to function in a CBF-independent pathway to regulate cold acclimation and freezing tolerance [14]. HOS15 interacts with H4 and represses *RD29A* expression by facilitating H4 deacetylation through association with the *RD29A* promoter. To determine whether *TCF1* functions in the same way as *HOS15*, *RD29A* expression was analyzed in *tcf1-1* plants under cold treatments. However, there were no changes in *RD29A* transcript levels detected in *tcf1-1* plants under stress treatment (Fig 4G). Importantly, in contrast to *hos15* the level of nuclear tetra-acetylated histone H4 in *tcf1-1* was similar to that in wild-type plants (Fig 4H). Together with the results of expression of *RD29A*, which is elevated in *hos15* plants in response to cold treatment [14] but not in *tcf1-1* (Fig 4G), we conclude that *TCF1* functions differently from *HOS15*.

### TCF1 Is Associated with Chromatin Containing the *BCB* Gene

**Fig 5:**
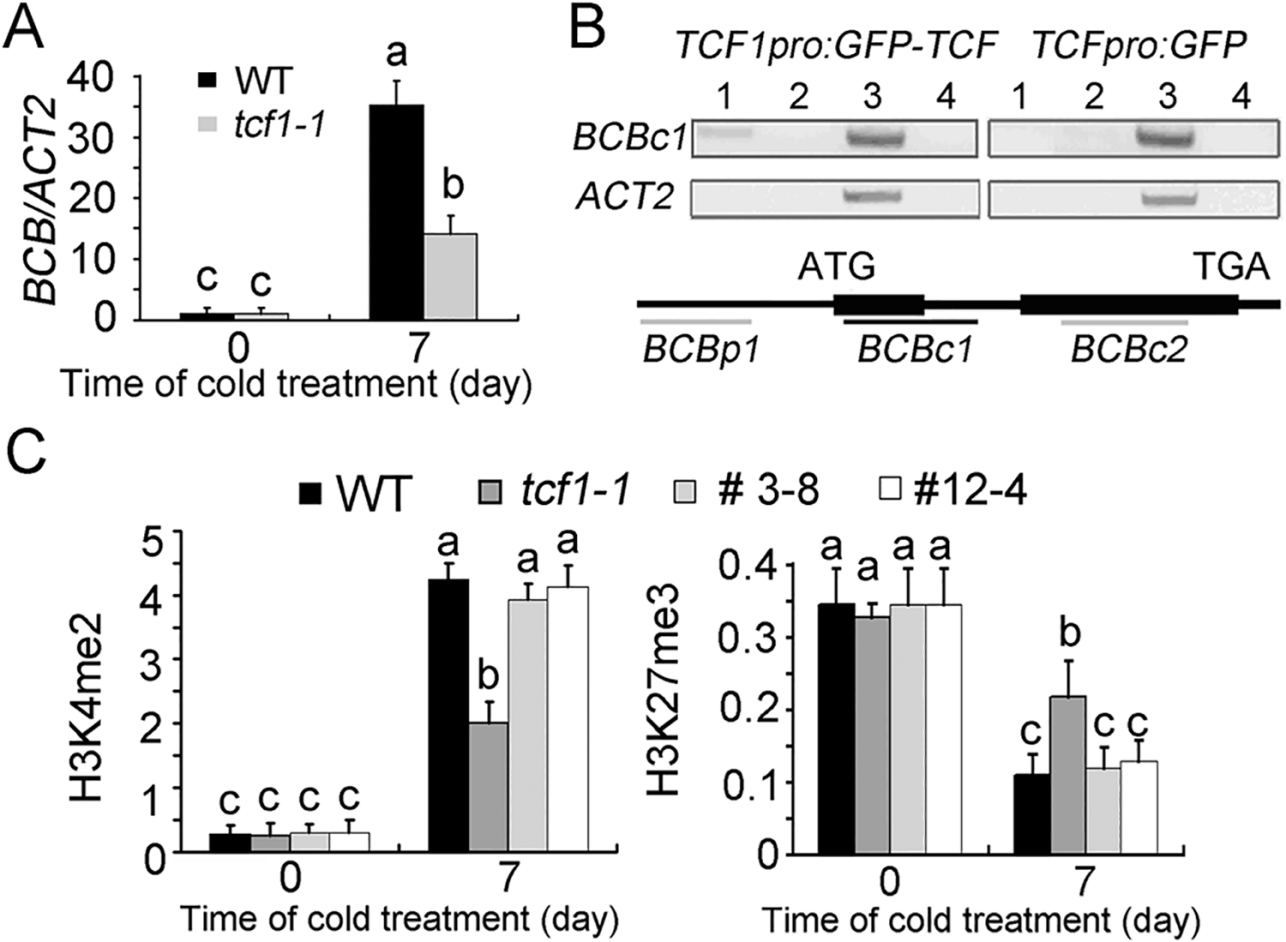
Chromatin immunoprecipitation assay of chromatin loci associated with TCF1. **(A)** The levels of *BCB* transcripts in *tcf1-1* (*Grey*) and wild-type (*Black*) plants. The plants were treated with cold at 4°C for 7 days. Data represent means of three replicates ± SD. Different Letters (*P* < 0.05; One Way ANOVA) show significant difference from WT. **(B)** ChIP assay for the association of TCF1 with *BCB* loci compared to the control *ACT2*. Lane 1, DNA immunoprecipitated by using anti-GFP antibody; lane 2, no antibody control; lane 3, input DNA before immunoprecipitation; lane 4, PCR without added DNA. *TCF1pro*::*GFP-TCF1* (Complemental line *tcf1-1TCF1-3-8*, *Left*) and *TCF1pro*::*GFP* (*tcf1-1Vector-2-3*, *Right*) transgenic plants treated at 4°C for 4 day were used in the experiments. *Below*: schematic presentation of the gene structures and the positions of the PCR fragments amplified from the ChIP products. Three fragments covering the genomic sequences of *BCB* (*BCBp1*, -664bp-301bp; *BCBc2*, 87bp-347bp; and *BCBc3*, 552bp-680bp) were used in the ChIP assay. The experiments were repeated three times; each gave similar data and results from one experiment are shown. **(C)** Levels of H3K4me2 and H3K27me3 by ChIP-qPCR analysis with WT, *tcf1-1* and two *TCF1* complementary transgenic lines (*tcf1-1TCF1-3* and *tcf1-1TCF1-12*) plants normalized to *ACT2* or *AGAMOUS* in *BCBc1* fragment with and without a 7-day cold treatment; data represent means of three biological replicates ± SD. Means with the same letter are not significantly different at *P* < 0.05 by One Way ANOVA analysis.

To investigate whether TCF1 represses or activates the expression of target genes through direct interaction with chromatin, we performed chromatin immunoprecipitation (ChIP) assay using *TCF1* complementational line (*tcf1-1TCF1-3*) that were cold-treated for 7 days. Seven genes (*At1g23150*, *At1g69120*, *At1g75040*, *At5g50720*, *At5g10760*, *At5g20230* and *At2g22500*), which were responsive to cold in *tcf1-1* in the microarray dataset were selected and three pairs of primers covering the entire genomic sequences of the candidate genes were designed. Using ChIP assay with an anti-GFP antibody, we found that among the tested genes, TCF1 was only associated with a chromatin fragment containing the coding region (*BCBc1*, CDS sequence +87 to +347) of the (*BLUE-COPPER-BINDING PROTEIN*) *BCB* gene (*At5g20230*), which is cold inducible (Fig 5A), whereas *ACT2* whose expression level is not changed in the mutant was not immunoprecipitated (Fig 5B). The results suggest that the association of TCF1 with chromatin at the *BCB* locus mediates cold-induced and TCF1-regulated expression of *BCB* gene.

### Histone Methylation May Be Involved in Cold Regulation of Transcription

To get a better understanding of the relationships between histone modifications and *BCB* gene expression, we analyzed histone modifications across the *BCB* locus in non-stressed and stressed wild-type, *tcf1-1* and *TCF1* complementational lines (Figs 5C and S7A). As histone H3K4me2 has been demonstrated to play widespread roles in activation of gene expression, we first analyzed the H3K4me2 status of the *BCB* gene. In the wild-type and *TCF1* complementation lines, *BCB* was induced by cold treatment. Accordingly, the level of H3K4me2 increased in the transcribed region of *BCB* (*BCBc1* fragment) after cold treatment (Figs 5C and S7A). Under normal condition, *tcf1-1* had comparable level of H3K4me2 to that of the wild-type. When exposed to cold for 7 days, *tcf1-1* also exhibited an increase in H3K4me2, but the level of H3K4me2 at the *BCB* locus was lower than that of the wild-type and *TCF1* complementation lines (Figs 5C and S7A).

Trimethylation of histone H3 at lysine 27 (H3K27me3) is a histone mark associated with gene silencing. We then analyzed the status of H3K27me3 at the *BCB* locus (*BCBc1* fragment) in non-stressed and stressed wild-type, *tcf1-1* and *tcf1-1TCF1* seedlings. In the non-stressed wild-type and *tcf1-1TCF1* lines, the level of H3K27me3 was relatively high. However, the level of H3K27me3 was significantly decreased when the seedlings were treated at low temperature for 7 days (Figs 5C and S7A). In sharp contrast, loss of *TCF1* function caused an opposite trend in H3K27me3. Under normal condition, the *tcf1-1* mutant displayed a similar level of H3K27me3 compared with that of the wild-type and *TCF1* complementational lines (Figs 5C and S7A). As expected, the level of H3K27me3 at the *BCBc1* region of *BCB* was higher in mutant compared with wild-type when exposed to low temperature for 7 days. We also tested H3K36me3 and H3K9me2 (for gene repression), H3K14ac and H3K9ac (for gene activation) and AcH4 (global histone H4 tetra-acetylation at K5/K8/K12/K16, which is also associated with gene activation) levels in the *BCBc1* fragment and found there were no significant differences between WT and *tcf1-1* plants (S7B–S7E Fig). The results indicate that TCF1 may regulate freezing tolerance of plants through modulation of histone modification and subsequent expression of target genes.

### Knock-down of the *BCB* Gene Results in Reduced Lignin Accumulation and Increased Cold Tolerance

**Fig 6:**
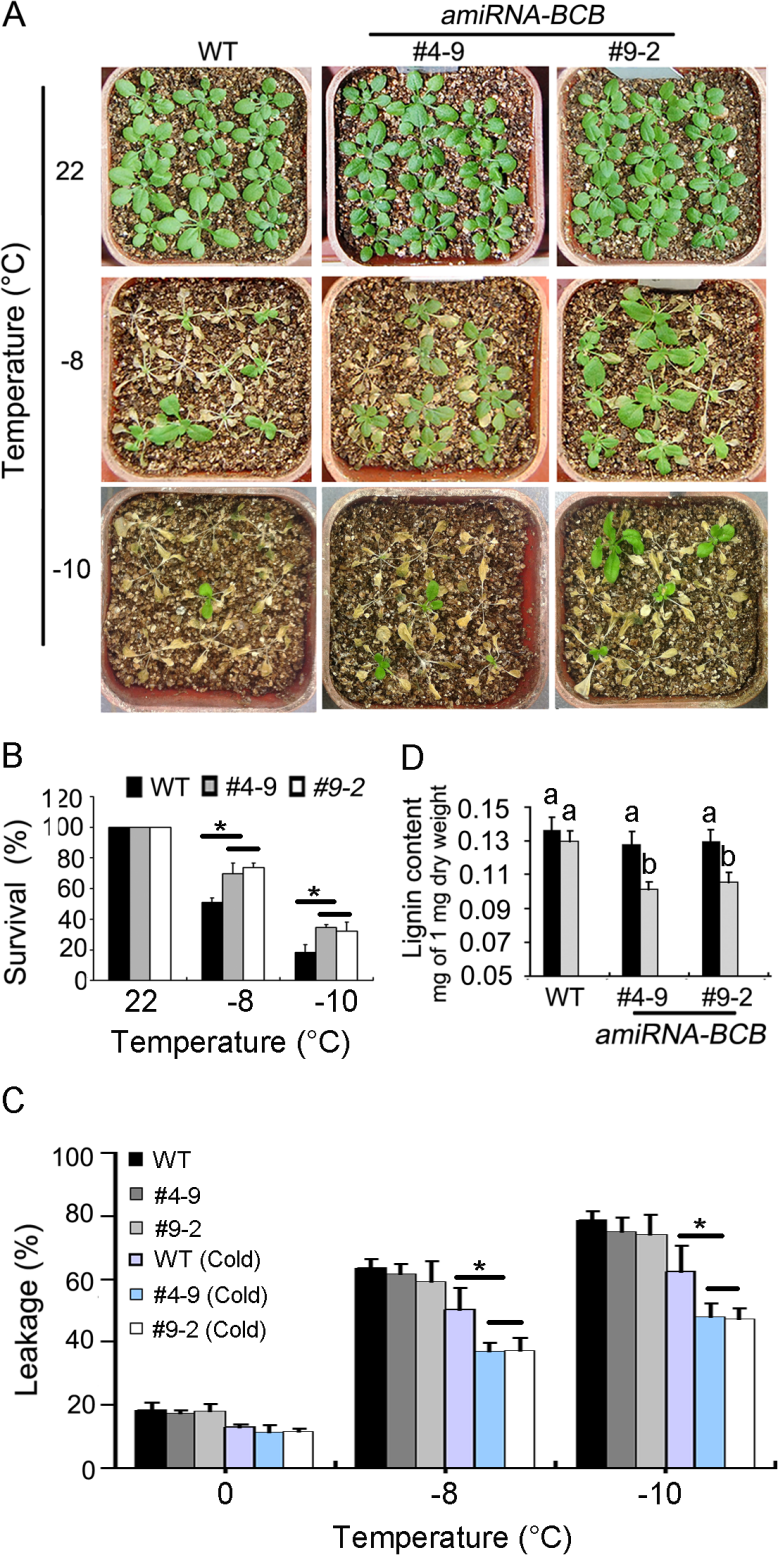
*amiRNA-BCB* plants are tolerant to freezing treatment. **(A)** Freezing treatment of 3-week-old *amiRNA-BCB* transgenic plants (#4–9 and #9–2) and wild-type (WT) plants at the indicated temperature below freezing under long-day photoperiod with cold acclimation. The pictures were taken 7 days after treatments. **(B)** Quantification of survival of the plants in **(A)**. Error bars are standard deviation (*n* = 80–100), (*, *P* < 0.05, *t*-test). **(C)** Leakage of electrolytes in *amiRNA-BCB* transgenic plants and WT plants treated at indicated temperature below freezing. Error bars are standard deviation (*n* = 8). (*, *P* < 0.05, *t*-test). **(D)** Quantitative determination of lignin content from whole rosettes of three-week-old WT and *amiRNA-BCB* transgenic plants grown in soil with (*Grey*) or without (*Black*) cold treatment (4°C for 7 days). Twelve independent experiments were performed and the data are expressed as mean ± S.E. Means with the same letter are not significantly different at *P* < 0.05 by One Way ANOVA analysis.

To test whether *BCB* is a functional target of TCF1 in plant response to cold, we used artificial microRNA (amiRNA) method to generate the *BCB* knock-down transgenic plants. Two transgenic lines with similar reduction in *BCB* expression to *tcf1-1* mutant (named *amiR-BCB4-9* and *amiR-BCB9-2*) (S8 Fig) were used for phenotypic analysis. Three week-old seedlings of the *BCB* knock-down lines and the wild type control with cold acclimation were treated at -8°C and -10°C for 2 h and were then grown under normal conditions for 7 days. The results showed that the survival rates of the *amiR-BCB* transgenic lines were markedly increased compared with that of the wild type (Figs 6A and 6B and S9A and S9B). The electrolyte leakage assay revealed that knock-down of *BCB* significantly reduced the electrolyte leakage from the treated plant cells (Fig 6C), confirming that *BCB* gene plays an important role in freezing tolerance of *Arabidopsis* plants during cold acclimation. However, we noticed that the average survival rate for the *amRNAi-BCB* lines (about 35%) is significantly lower than the *tcf1-1* (45.5%) under -10°C treatment (Figs 3D and 6B), indicating that in addition to *BCB*, alteration of other genes may also contribute to the freezing tolerance of *tcf1-1*.

The immediate question is how *BCB* regulates plant freezing tolerance. Since a previous study has shown that overexpression of *BCB* results in increased lignin accumulation in *Arabidopsis* [49], we attempted to test whether *BCB* regulates plant freezing tolerance through modification of lignin content of plants. To this end, we analyzed the lignin levels of 3-week-old seedlings of the *amiRBCB* transgenic lines and the wild-type that were treated at 4°C for 7 days. As shown in Fig 6D, the lignin content of the wild type seedlings was not affected by cold treatment, by contrast, the lignin levels of the *amiRBCB* transgenic lines was significantly reduced compared with WT under cold treatment (Fig 6D). The results suggest that *BCB* is responsible for maintaining steady lignin content in *Arabidopsis* plants under cold stress conditions. Taken together, these results indicate that TCF1 may modulate freezing tolerance through a *BCB*-dependent mechanism that positively regulates lignin biosynthesis.

### *TCF1* and *BCB* Modulate *PALs* Genes Expression to Affect Lignin Accumulation under Cold Acclimation

**Fig 7:**
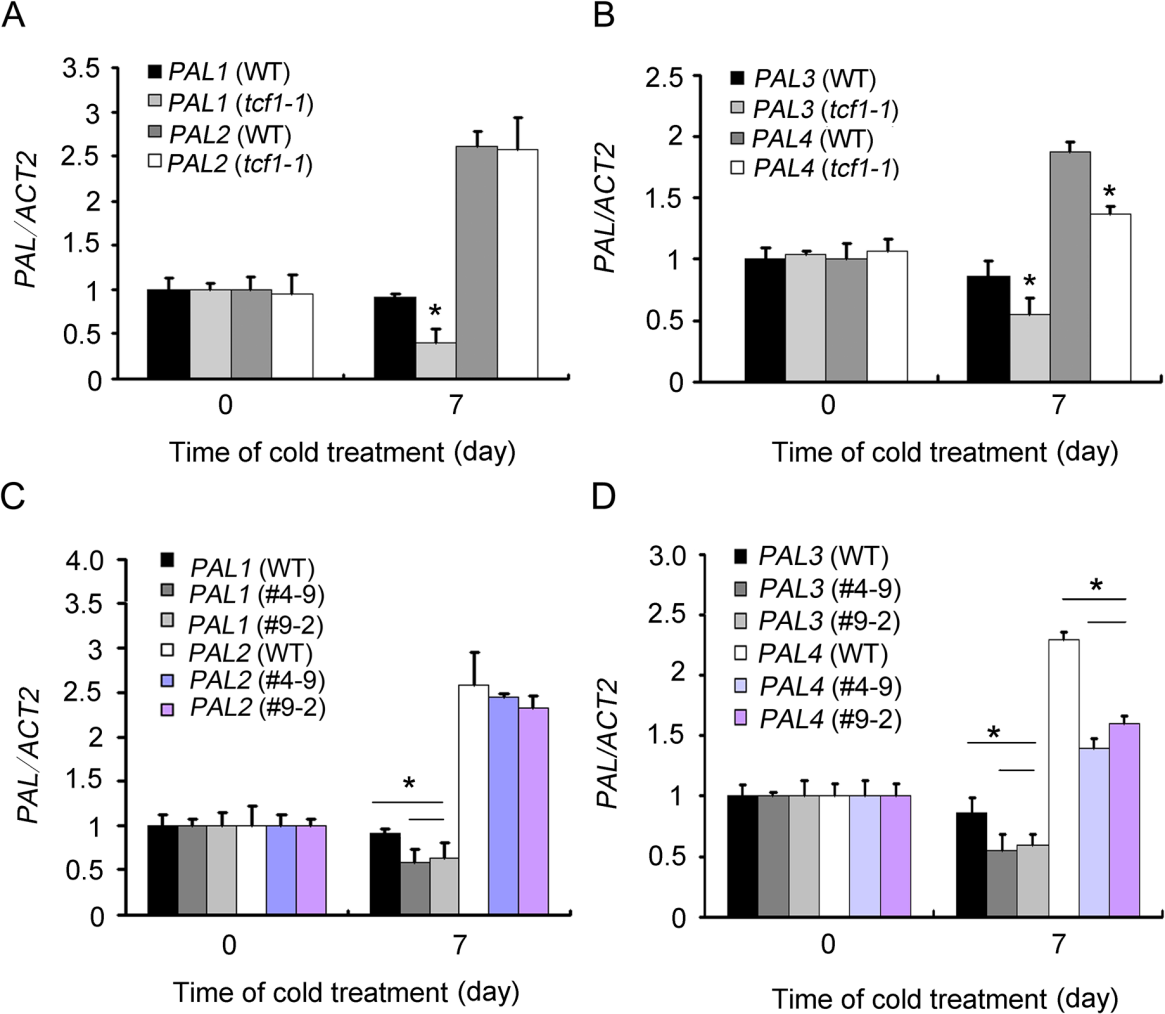
Expression analysis of *PAL* genes in *tcf1* and *amiRNA-BCB* mutants. **(A)** to **(D)** Quantitative RT-PCR analysis of the expression of *PALs* in three-week-old WT, *tcf1-1* and *amiRNA-BCB* plants grown in soil with or without cold treatment (4°C for 7 days). Three independent experiments were performed and the data are expressed as mean ±.SE. (*, *t*-test, *P* < 0.05).

To investigated whether the genes responsible for lignin biosynthesis showed any differential expression in *tcf1* in response to cold treatment. The transcript levels of *PAL* genes, which encode isoforms of a key enzyme Phe ammonia lyase involved in lignin biosynthesis were analyzed. In wild-type seedlings, the transcript levels of *PAL1* and *PAL3* remained not changed after cold treatment, but the *PAL2* and *PAL4* gene was induced by cold treatment (Fig 7A and 7B). In the *tcf1-1* mutant, expression of *PAL1*-*4* was similar to that of wild type under normal condition, but *PAL1*, *PAL3* and *PAL4* exhibited significantly decreased transcript accumulation after cold treatment. *PAL2* showed a similar level of transcript in the non-stressed and stressed *tcf1-1* mutant to that of the wild-type (Fig 7A and 7B).

We also checked the *PALs* expression in *amiRNA-BCB* transgenic lines, we found that *PAL2* showed a similar level of transcript in the non-stressed and stressed *amiRNB-BCB* mutant to that of the wild-type, but *PAL1*, *PAL3* and *PAL4* exhibited significantly decreased transcript accumulation after cold treatment in the *amiRNB-BCB* mutant (Fig 7C and 7D).These results suggest that *TCF1* and *BCB* positively regulates lignin content in rosettes under low temperature and that this may be due to regulation of genes involved in lignin biosynthesis.

### Reduced Lignin Content in *tcf1-1* Results in Freezing Tolerance Phenotype

**Fig 8:**
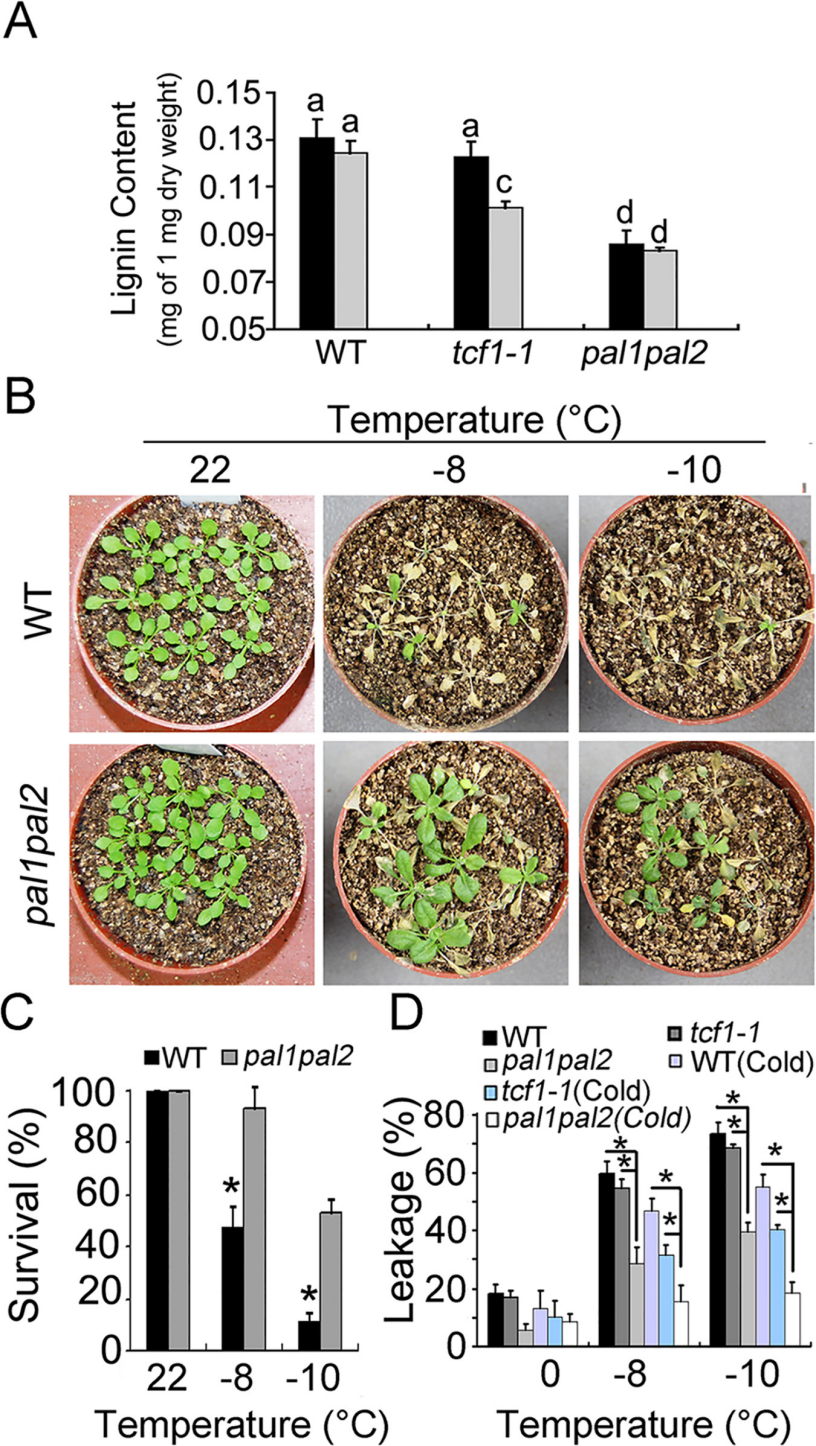
Reduced lignin content increased freezing tolerance. **(A)** Quantitative determination of lignin content from whole rosettes of three-week-old WT and *tcf1-1* plants grown in soil with (*Grey*) or without (*Black*) cold treatment (4°C for 7 days). Twelve independent experiments were performed and the data are expressed as mean ±S.E. Means with the same letter are not significantly different at *P* < 0.05 by One Way ANOVA analysis. **(B)** Three week-old *pal1pal2* and wild type (WT) plants with or without a 7-day cold treatment at 4°C were used for freezing treatments at indicated time point. The pictures were taken 7 days after treatments. **(C)** Quantification of survival of the wild type (*Black*) and *pal1pal2* plants (*Grey*) in **(B)**; Error bars represent standard deviation (*n* = 80–100). (*, *t*-test, *P* < 0.05). **(D)** Leakage of electrolytes in *pal1pal2* and wild type plants treated (see experimental procedures) at indicated time point with and without cold acclimation. The experiments were repeated 3 times. Error bars are standard deviation (*n* = 6). (*, *t*-test, *P* < 0.05).

To test whether TCF1 regulates plant freezing tolerance through modulating lignin biosynthesis, we measured the lignin contents in *tcf1* and wild-type plants with cold treatments. As expected, at the end of a 7-day cold treatment, the *tcf1* rosettes accumulated a significantly lower level of lignin compared with the wild-type (Fig 8A), suggesting a role of TCF1 in lignin biosynthesis.

As the loss of function in *TCF1* causes reduced lignin content and increased freezing tolerance, we proposed that under freezing temperature, reduced lignin may protect plant cells from freezing stress. To test this possibility, we analyzed the freezing tolerance of the *pal1pal2* (*pal1-2*/*pal2-2*) double mutant with or without cold acclimation. Under normal condition, the *pal1pal2* rosettes contained the lowest level of lignin reduction compared with the wild-type and *tcf1-1* mutant (Fig 8A). The substantial low level of lignin in the *pal1pal2* double mutant observed in this study is consistent with previous reports [56]. Based on our hypothesis, the less lignin the plants accumulate, the higher freezing tolerance they have. Similar sized wild-type and *pa11pal2* seedlings were subjected to freezing treatment with or without cold acclimation. As expected, the *pal1pal2* mutant displayed the highest freezing tolerance (Fig 8B–8D and S10A and S10B). The stronger effect of *pal1pal2* mutant on freezing tolerance than *tcf1* and wild-type suggests that the reduced lignin level is correlated to plant freezing tolerance.

## Discussion

The present study reports isolation and functional characterization of a novel nuclear protein TCF1 as a determinant of cold acclimation and freezing tolerance. We show that *TCF1* specifically activated by cold associates with chromatin and regulates a specific set of genes that are involved in cold acclimation and adaptation to freezing temperature. Importantly, TCF1 regulates lignin accumulation during acclimation/freezing and affects plant freezing tolerance via a CBF independent pathway. Thus, our study not only identifies a novel TCF1-mediated signaling cascade that plays a key role in cold acclimation and freezing tolerance, but also reveals a critical role of cell wall remodeling, in particular lignin homeostasis in cell wall in protecting cells from freezing damage.

TCF1 specifically responded to cold stress via both transcript and protein accumulation (Fig 1), and *tcf1-1* plants displayed specific enhancement of freezing tolerance after cold acclimation (Fig 3C and 3D). These results suggest that TCF1 is functions as a negative regulator in cold acclimation and freezing tolerance in *Arabidopsis*. Recently, extensive attention has been paid to the CBF signaling pathway, however, several studies have shown that the CBF signaling pathway is not the sole mechanism modulating plant cold acclimation and cold tolerance, because at least 28% of the cold-responsive genes were not regulated by the CBFs [12]. TCF1 may act independently of the CBF-COR signaling pathway, because expression of the *CBF* genes and CBF-regulated genes was unaltered in *tcf1-1* (Fig 4A–4C, S1 and S2 Tables), and *TCF1* expression remained unchanged in *cbf2* (Fig 4D) and in the CBFs-overexpression plants [12]. The *TCF1*-mediated pathway is also distinct from HOS15, because in *tcf1-1*, cold induction of *RD29A* and the level of nuclear tetra-acetylated histone H4 were not affected (Fig 4G and 4H). Thus, we conclude that TCF1 regulates plant cold acclimation and tolerance through at least one additional regulatory pathway. Further genetic analysis will be necessary to determine the genetic relationship between TCF1 and CBFs under cold acclimation and freezing tolerance.

TCF1 belongs to a family of RCC1-like proteins in *Arabidopsis*. RCC1 functions as the GEF for the small G-protein Ran and is critical for maintaining the RanGTP/RanGDP gradient across nuclear envelope [57]. Our data show that unlike RCC1, TCF1 had very low Ran-GEF activity (Figs 2D and S2), indicating that TCF1 is not the ortholog of RCC1 in *Arabidopsis*. However, TCF1 shares several important features with UVR8, another RCC1 family protein: very low GEF activity, function in nucleus (Fig 1E) and histone/chromatin association (Fig 2A–2C), although UVR8 preferentially interacts with histone H2B [40,58]. UVR8 regulates plant responses specifically to UV-B through modifying expression of *HY5* [40]. Thus, we hypothesized that TCF1 may regulate cold acclimation and freezing tolerance through a similar regulatory mechanism. Indeed, we found that TCF1 directly interacted with the coding regions of *BCB* (Fig 5B). Further we show that activity of *BCB* correlates with the enrichment of the positive mark H3K4me2 and reduction of the repressive mark H3K27me3 as cold acclimation is initiated (Figs 5C and S7A). Most importantly, TCF1 is required to modulate levels of both H3K4me2 and H3K27me3 at the *BCB* locus and regulate *BCB* transcription (Figs 5C and S7A). Therefore, H3K4me2 and H3K27me3 appear to synergistically regulate transcription activation of *BCB*, pointing to a critical role of active and repressive marks in cold acclimation.

It is clear that cold activates *TCF1* to induce or repress a set of target genes through a chromatin based mechanism. Interestingly, *BCB* encodes a Blue Copper Binding protein, which is a glycosylphosphatidylinositol-anchored protein (GAP) targeted to the cell surface [59,60] and seems to be responsible for lignin accumulation and cell wall-based resistance to aluminum and bacteria [49,61]. Therefore, our data support the hypothesis that *TCF1* regulates cold acclimation and freezing tolerance through modulating *BCB* to adjust lignin accumulation and consequent cell wall remodeling (Fig 6). We observed a *TCF1* dependent reduction in *BCB* expression and lignin content in rosette leaves of *tcf1-1* during cold acclimation (Fig 8A). Importantly, we found that expression of *PAL1*, *PAL3* and *PAL4* was reduced under cold in *tcf1-1* and *BCB* knock-down transgenic lines (Fig 7), suggesting that the transcriptional activity of the genes in leaves is also influenced by *TCF1* and *BCB*. Our data reveals that *TCF1* directly influences *BCB* activity and affects *PAL1*, *PAL3* and *PAL4* expression and lignification, although it is unclear whether *BCB* directly regulates *PAL* genes and lignification or whether *TCF1* mediated alteration of chromatin state during cold acclimation indirectly affects *PALs* expression. Therefore, our data define TCF1 as a key factor able to bind to chromatin and epigenetically regulate the cold-specific GAP leading to establishment of cold specific transcriptional programs and consequently lignin and extracellular matrix remodeling. Although *BCB* level was almost the same in *tcf1-1* and *amiRNA-BCB* lines, *tcf1-1* showed higher survival rate than *amRNAi-BCB* lines under freezing treatments (Figs 3D and 6B), pointing out that *BCB* was not the only gene to involve in *TCF1*-mediated freezing tolerance. Further identification of the target gene(s) of TCF1 will help us to uncover the molecular mechanism underlying TCF1-mediated plant cold acclimation and freezing tolerance. Since UVR8 can also modulate plant response to UV-B through direct interaction with COP1 [62]. We do not exclude the possibility that TCF1 regulates cold acclimation and freezing through association with the key regulator(s) in the cold signal transduction pathway.

It is well known that lignin fills the spaces in the cell wall to reduce water permeability and increase the stiffness of the cell wall [45,50]. Extensive cellular studies have shown that freezing tolerance is directly related to cell permeability and cell wall properties, in particular lignin content, so that water outflows and ice forms in the extracellular spaces without damaging cellular structures [18,35,63–65]. Although it is still technically difficult to measure the cell wall permeability of plants during cold acclimation, the important role of lignin in plant cold acclimation and freezing tolerance has been well documented. For example, the freezing tolerant *Miscanthus* contains lower lignin content and higher *PAL* gene expression than the freezing sensitive ecotype [66]. Therefore, it is conceivable that lignin content is closely related to the cell wall permeability and freezing tolerance. However, the molecular mechanism by which lignin content is regulated during cellular adaptation to low temperature still remains a mystery. Here we demonstrate that during cold acclimation *tcf1-1*, *amiRNA-BCB* and *pal1pal2* had increased freezing tolerance which correlates with their reduced lignin contents (Figs 6 and 8). Therefore, reduction of lignin deposition within the cell wall of the *tcf1-1*, *amiRNA-BCB* and *pal1pal2* plants during acclimation and freezing may increase cell wall permeability and protect the cells from freezing damage. Reduction of lignin may also enhance elasticity of the cell wall to increase the capacity to accommodate growth of ice crystals with less damage to both the dehydrated cell and cell wall. Together, our data reveal a novel regulatory mechanism in cold acclimation and freezing tolerance in *Arabidopsis* that involves chromatin based regulation of lignification and cell wall remodeling.

**Fig 9:**
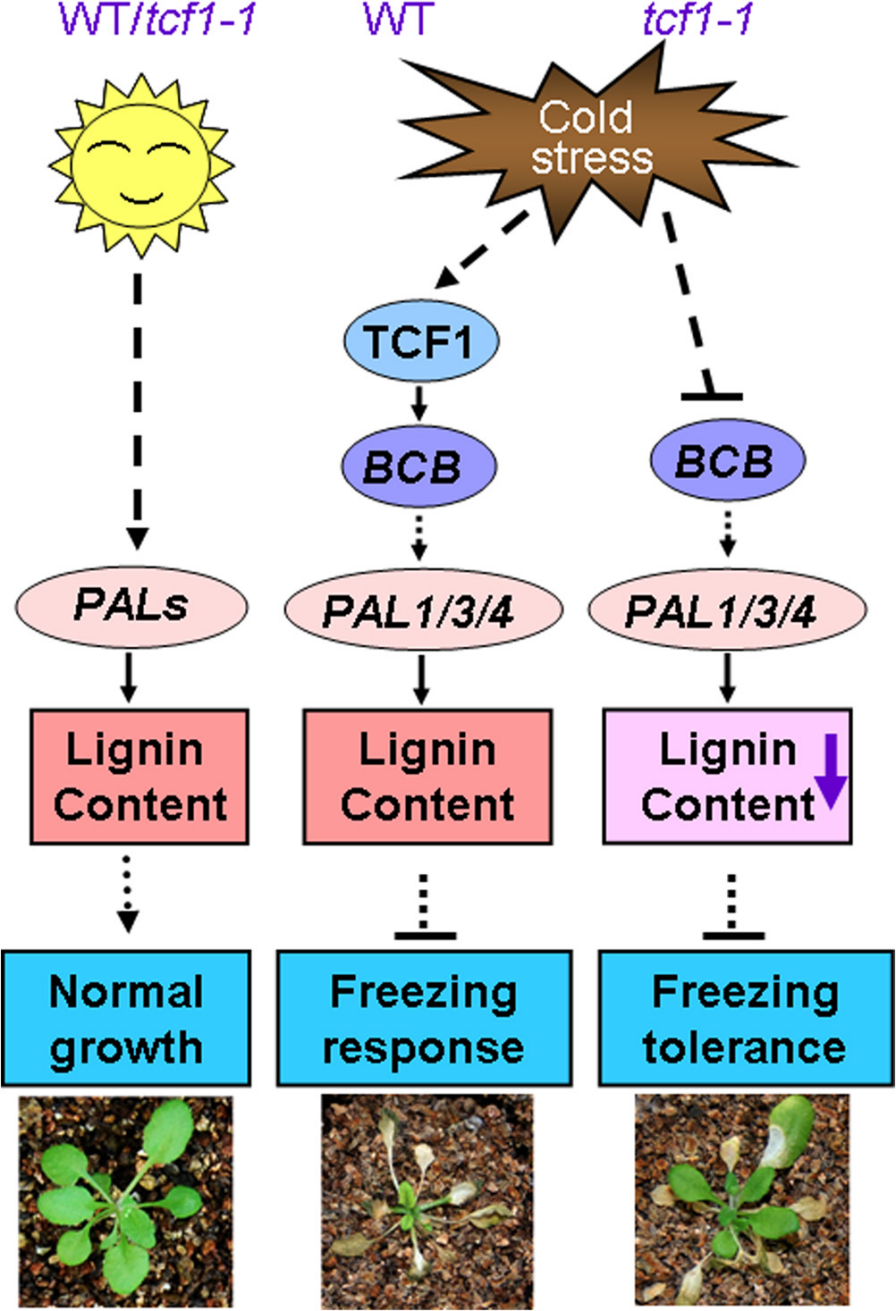
A proposed model for TCF1 in the *Arabidopsis* response to low temperature. Under normal condition, *PAL* genes are modulated by the developmental signals to synthesize lignin that favor optimal plant growth and water transport. During cold acclimation, *TCF1* is rapidly induced to activate *BCB* transcription and then stimulates expression of *PAL1*/*3/4* genes to maintain lignin accumulation of the stressed cells. However, when the plants are exposed to freezing temperature, reduction of lignin deposition within the cell wall of the *tcf1-1* plants may increase cell wall permeability and protect the cells from freezing damage. Reduction of lignin may also enhance elasticity of the cell wall to increase the capacity to accommodate growth of ice crystals with less damage to both the dehydrated cell and cell wall which is required for plant growth arrest. Lines with arrowheads denote direct regulation, and lines with blunt heads represent indirect regulation.

The immediate question is what the biological significance of TCF1 induction is because absence of TCF1 confers freezing tolerance. It is known that cold hardy (freezing tolerant) plants frequently employ extracellular freezing to cope with the freezing temperature. Arabidopsis Col-0 is not hardy plant although it has moderate freezing tolerance compared with other ecotypes [67]. It is possible that TCF1-mediated signaling is activated to maintain lignin content of cells that can enhance cell rigidity and reduce cell expansion, which is required for plant growth arrest under low temperature. Thus, it is conceivable that TCF1-mediated signaling modulates plastic development of the plants during cold acclimation, but not freezing tolerance. A mechanistic working model is presented in Fig 9. The fact that absence of TCF1 enhances freezing tolerance of Col-0 plants suggests that low expression or TCF1 absence may be related to plant freezing tolerance. Thus, our study also identifies a new gene that can be used for genetic improvement of plant freezing tolerance. It will be interesting to see whether *TCF1* gene acts differently in cold hardy plants. Given that natural variation in TCF1 may contribute substantially to cold acclimation and freezing tolerance among *Arabidopsis* accessions, the role of *TCF1* expression and its mediated signaling in cold acclimation and freezing tolerance is worthy of special focus. Furthermore, further study will also help to differentiate the molecular mechanisms in plant cold acclimation and freezing tolerance.

## Materials and Methods

### Plant Materials and Freezing Treatments

*A*. *thaliana* (L.) Heynh Col-0 and *tcf1-1* (SAIL_97_D05) and *tcf1-2* (SAIL_749_A09) from ABRC were germinated on MS medium containing 2% sucrose, pH 5.7. Three-week-old *tcf1-1* and wild-type plants grown in soil with cold-acclimation (4°C for 7 days) and non-cold acclimation were used for whole plant freezing tolerance tests and an electrolyte leakage assay (ELA) as previously described [13]. Briefly, the plants were grown in soil under a long-day photoperiod (16 h light/8 h dark) for 3 weeks in a growth chamber (22°C), and then placed in a temperature chamber (ZSP-A0160, ZHCHENG) at -6°C, -8°C or -10°C for 2 hours. Freezing tolerance was determined as the capacity of plants to resume growth after 7 days under control conditions. For ELA, the rosettes were placed in a temperature chamber starting at 0°C. The temperature was reduced by 2°C after 30 min and maintained for 1 h. Then an identical timing sequence was used for successive 2°C decreases until -10°C was reached. The percentage of EL was calculated as the percentage of the conductivity before autoclaving over that after autoclaving.

### Gene Expression and Microarray Analysis

Total RNA was extracted from plants’ leaves by using the TRizol Reagent. First-strand cDNA synthesis was performed according to standard procedures using reverse transcriptase (Promega, 18064–014) following the manufacturer’s instructions. Semi-quantitative RT-PCR and qRT-PCR were done as described previously [68]. A positive control was provided by a parallel analysis based on the *ACT2* gene, and three independent replicates were performed per experiment. Gene-specific primers for *CBF1*, *CBF2*, *CBF3* etc are shown in S3 Table.

For Microarray analysis, 3-week-old plants were treated with or without low temperature at 4°C for 7 day, 2 μg of total RNA was used to produce cyanine dye-tagged cRNA (cy5-WT, cy3-*tcf1*) and was hybridized to JingXin Array (CapitalBio Company) containing 29K *Arabidopsis* transcripts. Three biological replicates with 6 Microarray slides were used to check differential expression genes. Data from the GeneChip arrays were scanned on a GeneChip Scanner 3000 and analyzed using GeneChip Operating software (GCOS 1.4). The Significant Analysis of Microarray software (SAM) was used to identify significantly differentially expressed genes between *tcf1* and WT groups. Different genes were determined to be significantly differentially expressed with a selection threshold of false discovery rate, FDR = 5% and fold change > 1.5 in the SAM output result. The Microarray data had submitted to GEO (Accession number: GSE70682) at NCBI website.

### Plasmid Constructs and Transformation

For histochemical analysis of *TCF1* expression, a genomic fragment including 1,337 bp upstream of the translation initiation codon was amplified by PCR and cloned into the binary vector pCAMBIA1391 between the *Hin*dIII and *Bam*HI sites. β-glucuronidase (GUS) activity was assayed as previously described [68]. To make the *TCF1pro*::*GFP-TCF1* fusion, the *TCF1* CDS was first amplified by PCR and cloned in-frame into the binary vector pEZR(K)-LC [40] between the *Eco*RI and *Sal*I sites, and then the *35S* promoter was replaced by the 1,337 bp *TCF1* promoter between the *Hind*III and *Sac*I sites to generate *TCF1pro*::*GFP-TCF1*. The construct was introduced into wild-type and/or *tcf1-1* plants through *A*. *tumefaciens-*mediated transformation. At least three independent homozygous T3 lines were tested for *TCF1pro*::*GUS* expression, protein subcellular localization, and gene expression analysis.

### Yeast Two-Hybrid Assays

The *TCF1* coding region was amplified by PCR and cloned in-frame between the *Eco*RI and *Bam*HI sites of pGBKT7. *HTA1* [histone H2A (AT5g54640)] and *HTR9* [histone H3 (AT5g10400)] coding regions were amplified and cloned in-frame into pGADT7 between the *Nde*I and *Xho*I sites to generate prey constructs. pACT2-HTB1 (histone H2B) and pACT2-HFO1 (histone H4) were from Dr. R. A. Bressan at Purdue University. For analysis of specific histone H4 interaction with TCF1, *TCF1* coding region was cloned in-frame between the *Eco*RI and *Bam*HI sites of pGADT7, another two histone H4 variants (At5g59690 and At1g07820) with high expression in *tcf1-1* were introduced into pGBKT7 at *Eco*RI and *Sal*I sites. Plasmid DNA of bait and prey constructs was transformed into the *S*. *cerevisiae* strain Y190. Individual transformants were streaked on plates containing a synthetic, minimal (SD) medium lacking tryptophan and leucine and grown for 24 h. Yeast cells were transferred onto a filter paper, and β-galactosidase (β-gal) filter assays were performed [14].

### Determination of Histone Acetylation

Nuclei were isolated as described by [14]. Twenty micrograms of nuclear protein and 1 μg of purified core histones from chicken (Upstate Biotechnology, 13–107) were separated by SDS/PAGE and blotted onto a PVDF membrane (Millipore, GVPPEAC12). Anti-tetra-acetylated-histone H4 (1:1,000) or anti-histone H4 (1:100,000) (Upstate Biotechnology) primary antibodies were used to detect acetylated and unacetylated histone H4, bands were visualized by using the BCIP/NBT Kit (Invitrogen). Data shown are representative of six independent experiments.

### GEF Activity Assays, Histone Interaction and Histone Competition

GEF activity assay was performed as described in [40]. Briefly, RCC1, TCF1, and human Ran were expressed in *Escherichia coli* as fusions with GST. The Ran clone was provided by Dr. Murray Stewart (Medical Research Council Laboratory for Molecular Biology, Cambridge, U.K.). Assays of guanine nucleotide exchange activity were performed by using [^3^H]GDP to load 30 pmol GST-Ran and subsequent incubation with 0.5 nM recombinant RCC1 or TCF1 for 3 min. The exchange activity was calculated as ln (*C*_*t*_/*C*_*0*_), where *C*_*0*_ and *C*_*t*_ are radioactive counts at the start and end of the reaction, respectively. The GEF assays were repeated four times. Analysis of TCF1-histone interaction was performed as previously described [40]. Competition assays between TCF1 and histones were conducted as previously described by Cloix and Jenkins [58].

### Chromatin Association

Chromatin was isolated and the chromatin immunoprecipitation assay was carried out [40,69] by using an anti-GFP antibody (Invitrogen A-11122). Before antibody treatment, the samples were precleared with protein A Dynabeads (Dynal Biotech, Great Neck, NY, 100.02). The immunoprecipitated DNA was used in PCR reactions to amplify fragments from the *BCB* and *ACT2* genes, using primers shown in S3 Table. Data shown are representative of three independent experiments.

### Lignin Analysis

Cell wall fractions were isolated as described by Tokunaga et al [70]. Briefly, three-week-old plants at rosette stage were ground in liquid nitrogen, and then washed with 95% ethanol and ethanol:hexane (1:2, v/v) in turn. The washed pellet was allowed to air-dry at 70°C overnight. Lignin content was measured according to the method of Fukuda and Komamine [71] with some modifications. Five mg of the air-dried samples suspended in a 1 ml aliquot of 25% acetyl bromide in acetic acid were treated at 70°C for 30 min. After cooling down, 0.9 ml of 2 M NaOH, 5 ml of acetic acid, 0.1 ml of 7.5 M hydroxylamine hydrochloride, and 3 ml of glacial acetic acid were added. The 10 ml samples were centrifuged and the absorbance of the supernatant was measured at 280 nm to determine the lignin content.

## Supporting Information

S1 Fig *TCF1* expression and computational analyses of TCF1 protein. **(A)** Arabidopsis eFP Browser online software (http://bbc.botany.utoronto.ca/efp/cgi-bin/efpWeb.cgi) shows the *TCF1* gene expression was induced by cold stress. **(B)** Clustal W2 analysis of identity between RCC1 and TCF1 protein (http://www.ebi.ac.uk/Tools/msa/clustalw2/). Arrows in different colors with letter ABCD stands marked seven RCC1 repeat domains. **(C)** Expasy online software (http://www.expasy.org/scanprosite) predicted that *Homo sapiens* RCC1 contains 7 RCC1 repeats. Using the same program, we found that the TCF1 protein contains six predicted RCC1 repeats that are located as follows: 83–135, 178–252, 253–329, 330–381, 382–435, and 436–486.
(TIF)

S2 Fig TCF1 protein does not complement the temperature-sensitive phenotype of *prp20* mutants. Yeast *prp20* mutant was transformed with the indicated vector. Individual colonies of transformants were streaked on SD plates lacking Ura with galactose as carbon source and put in 23°C /37°C for 24 h simultaneously. *prp20*+RCC1, *prp20* transformed with human *RCC1* gene. *prp20*+Vector, *prp20* transformed with empty vector pMB150. *prp20*+TCF1, *prp20* transformed with *TCF1* gene.
(TIF)

S3 Fig *tcf1-1* plants show the same phenotype with WT without cold acclimation. **(A)** Freezing analysis of 3-week-old *tcf1-1* and wild-type (WT) plants at the indicated temperatures below freezing under long-day photoperiod without cold acclimation. Quantification of survival rate was taken at 7th days after treatments. **(B)** RT-PCR analysis of *TCF1* expression in two *TCF1-RNAi* lines (*TCF1-RNAi-2* and *TCF1-RNAi-6*) at 4°C for 7 day. **(C)** Freezing analysis of 3-week-old *TCF1-RNAi* lines and wild-type (WT) plants at the indicated temperatures below freezing under long-day photoperiod without cold acclimation. Quantification of survival rate was taken at 7th days after treatments. **(D)** Tolerance of 3-week-old *TCF1-RNAi* lines (*TCF1-RNAi-2* and *TCF1-RNAi-6*) and wild-type (WT) plants at the indicated temperatures below freezing under long-day photoperiod with cold acclimation for 7 days. The pictures were taken 7 days after treatments. **(E)** Quantification of survival rate of the treated plants in **(C)**, (*, *P* < 0.05, *t*-test). **(F)** Leakage of electrolytes in *TCF1-RNAi* lines and WT plants treated at indicated temperatures below freezing. Error bars are standard deviation (*n* = 8), (*, *P* < 0.05, *t*-test).
(TIF)

S4 Fig *TCF1* gene complements the *tcf1-1* freezing tolerant phenotype. **(A)** The three week-old non-acclimated plants were treated at -8°C for 2 h followed by a seven day recovery. These include *tcf1-1*, WT, a representative line of *tcf1-1* transformed with an empty vector pEZR(K)LC (*tcf1-1Vector-2*), and three independent homozygous lines of *tcf1-1* transformed with *TCF1* gene (*tcf1-1TCF1-3*, *tcf1-1TCF1-12* and *tcf1-1TCF1-13*) without cold acclimation. **(B)** Semi-quantitative RT-PCR for *TCF1* expression in the wild type and transgenic plants. The three-week-old plants were treated with or without a seven-day cold acclimation at 4°C, respectively. Lane 1: WT, lane 2: *tcf1-1*, lane 3: *tcf1-1Vector-2*, lane 4: *tcf1-1TCF1-3*, lane 5: *tcf1-1TCF1-12*, lane 6: *tcf1-1TCF1-13*. **(C)** Quantification of survival rate of cold acclimated (black) and non-acclimated (grey) plants after freezing treatment at -8°C for 2 h and a seven day period of recovery. 1: WT, 2: *tcf1-1*, 3: *tcf1-1Vector-2*, 4: *tcf1-1TCF1-3*, 5: *tcf1-1TCF1-12*, 6: *tcf1-1TCF1-13*. Error bars are standard deviation (*n* = 80–100). Three biological experiments were performed and the data are expressed as mean ±S.E. Means with the same letter are not significantly different at *P* < 0.05 by One Way ANOVA analysis. **(D)** Leakage of electrolytes in WT, *tcf1-1* and *TCF1* complementary lines (*tcf1-1TCF1-3*, *tcf1-1TCF1-12* and *tcf1-1TCF1-13*) treated at indicated temperatures below freezing. 3-week-old plants were cold-acclimated at 4°C for 7 day (Cold) or without treatment. Error bars are standard deviation (*n* = 10), (*, *P* < 0.05, *t*-test).
(TIF)

S5 Fig Expression of *CBFs* in the wild-type and *tcf1-1* plants under treatment. **(A)** The three week-old *tcf1-1* and wild type plants were treated with cold at 4°C and rosettes were harvested at the indicated time points for RNA extraction and semi-quantitative RT-PCR. Rosettes of the plants treated with 100 μM ABA, 400 mM mannitol or 300 mM NaCl for 3 h were also collected for gene expression analysis. Transcript levels were assayed for *CBF* genes and loading control *ACTIN2*. **(B)** Semi-quantitative RT-PCR for *CBF2* expression in a T-DNA insertion line. Three-week-old SALK_025203 and Col-0 seedlings grown on soil were subjected to low temperature (4°C) for 3 h, and the shoots were collected for the expression analysis. *ACT2* gene was used as a loading control. The result showed that the mutant is a null mutation for the *CBF2* gene, and was named as *cbf2*.
(TIF)

S6 Fig Quantitative RT-PCR analysis of the *AT2G33810*, *AT1G23150*, *AT3G57260* and *AT3G51330* genes expression. Three-week-old WT and *tcf1-1* grown on MS medium plates were treated with cold stress for 7 day, and the levels of four genes’ transcripts were analyzed. The genes showed altered transcript levels in *tcf1-1* in the microarray analysis were selected. *ACT2* gene was used as a loading control. CA: Cold acclimation, NA: None cold acclimation.
(TIF)

S7 Fig Level of histone markers in wild-type, *tcf1-1* and two *TCF1* complementary lines (*tcf1-1TCF1-3* and *tcf1-1TCF1-12*) with or without cold acclimation. **(A)** Levels of H3K4me2 and H3K27me3 by ChIP-PCR analysis with wild-type (WT), *tcf1-1*, *tcf1-1TCF1-3* and *tcf1-1TCF1-12* plants normalized to *ACT2* or *AGAMOUS* in *BCBc1* fragment with and without a 7-day cold treatment. **(B)** to **(E)** Levels of H3K9me2, H3K9ac, H4Ac (The level of global histone H4 tetra-acetylation at K5/K8/K12/K16), H3K14ac and H3K36me3 by ChIP-PCR analysis normalized to indicated control in *BCBc1* fragment in wild type and *tcf1-1* plants with a seven-day cold acclimation.
(TIF)

S8 Fig Reduced *BCB* expression in *amiRNA-BCB* plants under cold acclimation. The levels of *BCB* transcripts in wild-type (*Black*) and *amiRNA-BCB* (*Grey*) plants. The plants were treated with cold at 4°C for 7 days. Three biological experiments were performed and the data are expressed as mean ±S.E. Means with the same letter are not significantly different at *P* < 0.05 by One Way ANOVA analysis.
(TIF)

S9 Fig *amiRNA-BCB* plants show similar freezing phenotype with WT without cold acclimation. **(A)** Freezing treatment of three-week-old *amiRNA-BCB* transgenic plants (#4–9 and #9–2) and wild-type (WT) plants at the indicated temperature without cold acclimation. The pictures were taken 7-days after treatments. **(B)** Quantification of survival of the plants in **(A)**. Error bars are standard deviation (*n* = 80–100).
(TIF)

S10 Fig *pal1pal2* is freezing tolerant compared with WT. **(A)** Three week-old *pal1pal2* and wild type (WT) plants without a 7-day cold treatment were used for freezing treatments at indicated time. The pictures were taken 7 days after treatments. **(B)** Quantification of survival of the wild type (*Black*) and *pal1pal2* plants (*Grey*) in **(A)**. Error bars represent standard deviation (*n* = 80–100).
(TIF)

S1 Table Genes with increased and decreased expression levels by 1.5-fold in *tcf1-1* without cold acclimation determined by Biocapital Jingxin microarray. (DOC)

S2 Table Genes with increased and decreased expression levels by 1.5-fold in *tcf1-1* with cold acclimation determined by Biocapital Jingxin microarray. (DOC)

S3 Table List of primers. (DOC)
