# Supplementary figures and images for: The Arabidopsis RCC1 Family Protein TCF1 Regulates Freezing Tolerance and Cold Acclimation through Modulating Lignin Biosynthesis
Source: PLoS Genet. 2015 Sep 22;11(9):e1005471. doi: 10.1371/journal.pgen.1005471 (PMC4579128; doi:10.1371/journal.pgen.1005471)

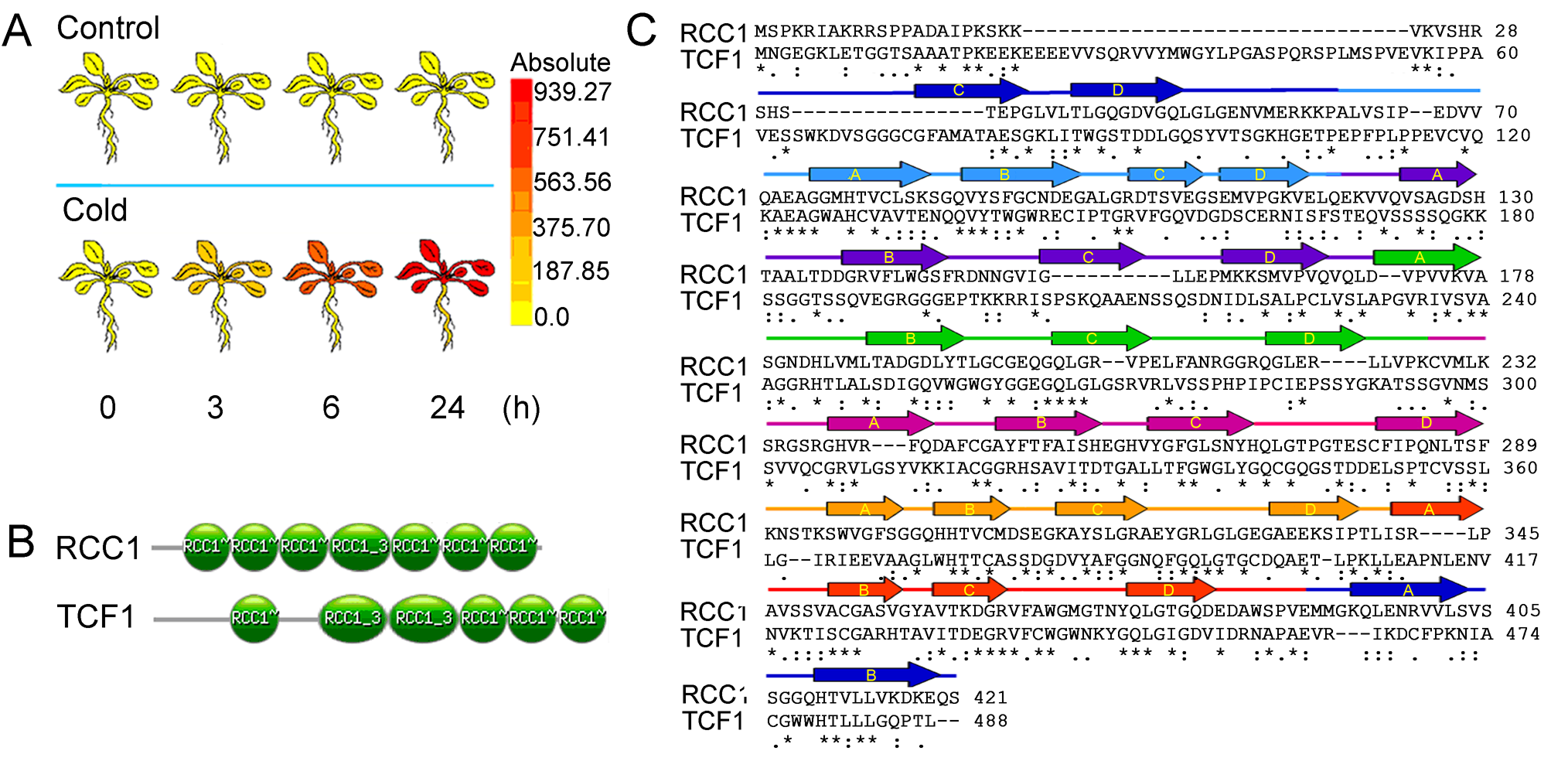

Supplement: S1 Fig — (A) Arabidopsis eFP Browser online software (http://bbc.botany.utoronto.ca/efp/cgi-bin/efpWeb.cgi) shows the TCF1 gene expression was induced by cold stress. (B) Clustal W2 analysis of identity between RCC1 and TCF1 protein (http://www.ebi.ac.uk/Tools/msa/clustalw2/). Arrows in different colors with letter ABCD stands marked seven RCC1 repeat domains. (C) Expasy online software (http://www.expasy.org/scanprosite) predicted that Homo sapiens RCC1 contains 7 RCC1 repeats. Using the same program, we found that the TCF1 protein contains six predicted RCC1 repeats that are located as follows: 83–135, 178–252, 253–329, 330–381, 382–435, and 436–486. (TIF) [file pgen.1005471.s001.tif]

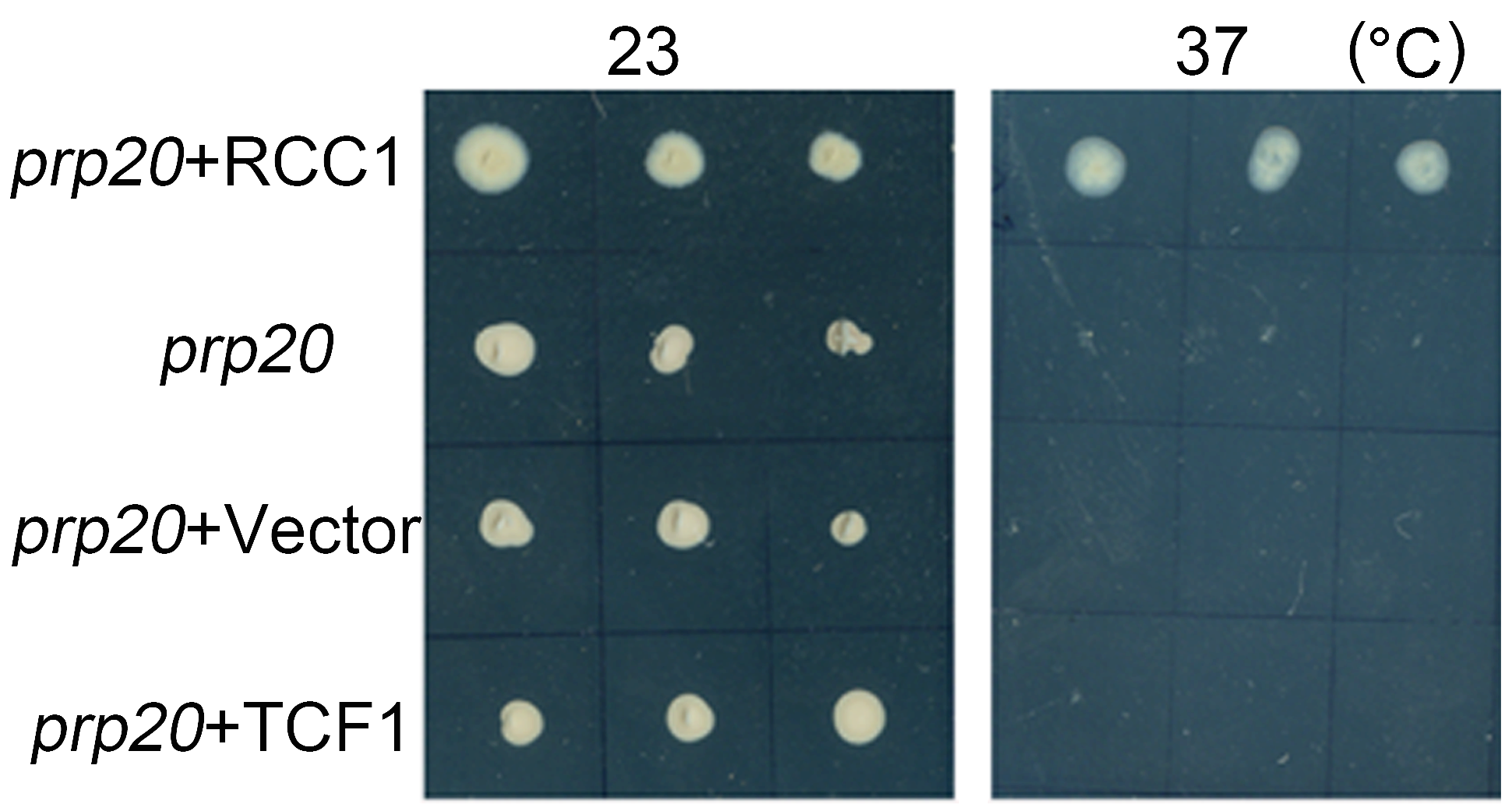

Supplement: S2 Fig — Yeast prp20 mutant was transformed with the indicated vector. Individual colonies of transformants were streaked on SD plates lacking Ura with galactose as carbon source and put in 23°C /37°C for 24 h simultaneously. prp20+RCC1, prp20 transformed with human RCC1 gene. prp20+Vector, prp20 transformed with empty vector pMB150. prp20+TCF1, prp20 transformed with TCF1 gene. (TIF) [file pgen.1005471.s002.tif]

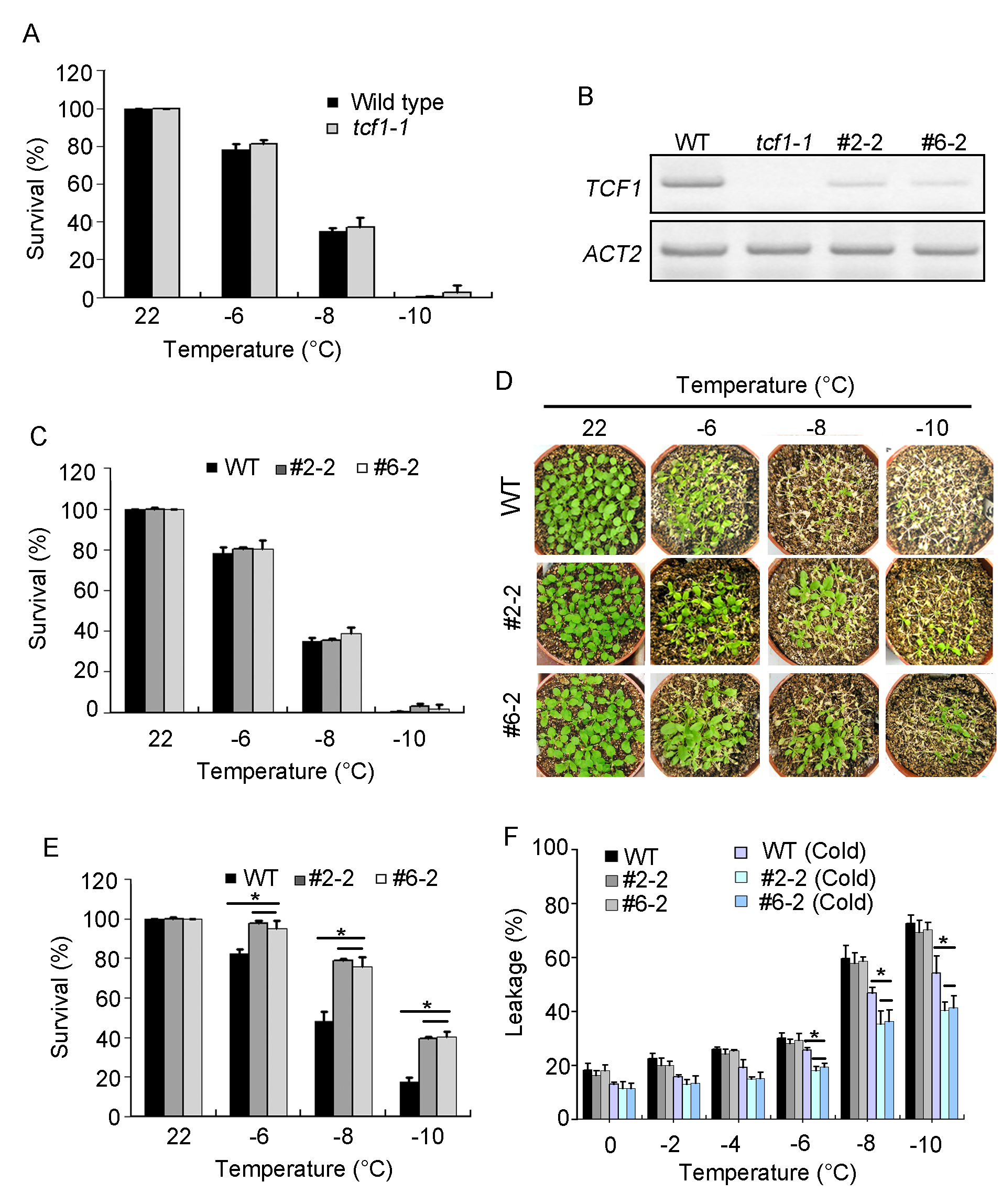

Supplement: S3 Fig — (A) Freezing analysis of 3-week-old tcf1-1 and wild-type (WT) plants at the indicated temperatures below freezing under long-day photoperiod without cold acclimation. Quantification of survival rate was taken at 7th days after treatments. (B) RT-PCR analysis of TCF1 expression in two TCF1-RNAi lines (TCF1-RNAi-2 and TCF1-RNAi-6) at 4°C for 7 day. (C) Freezing analysis of 3-week-old TCF1-RNAi lines and wild-type (WT) plants at the indicated temperatures below freezing under long-day photoperiod without cold acclimation. Quantification of survival rate was taken at 7th days after treatments. (D) Tolerance of 3-week-old TCF1-RNAi lines (TCF1-RNAi-2 and TCF1-RNAi-6) and wild-type (WT) plants at the indicated temperatures below freezing under long-day photoperiod with cold acclimation for 7 days. The pictures were taken 7 days after treatments. (E) Quantification of survival rate of the treated plants in (C), (*, P < 0.05, t-test). (F) Leakage of electrolytes in TCF1-RNAi lines and WT plants treated at indicated temperatures below freezing. Error bars are standard deviation (n = 8), (*, P < 0.05, t-test). (TIF) [file pgen.1005471.s003.tif]

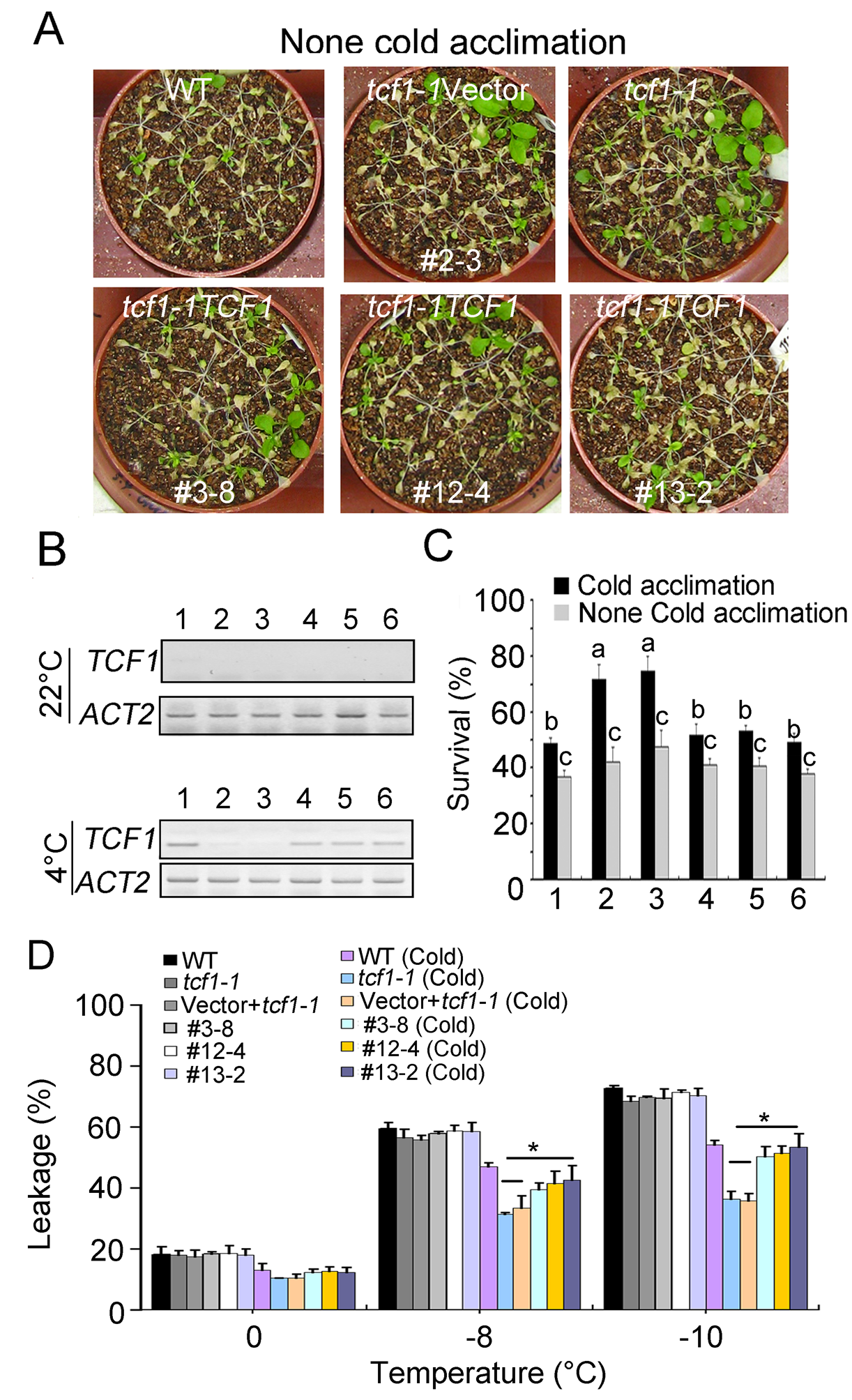

Supplement: S4 Fig — (A) The three week-old non-acclimated plants were treated at -8°C for 2 h followed by a seven day recovery. These include tcf1-1, WT, a representative line of tcf1-1 transformed with an empty vector pEZR(K)LC (tcf1-1Vector-2), and three independent homozygous lines of tcf1-1 transformed with TCF1 gene (tcf1-1TCF1-3, tcf1-1TCF1-12 and tcf1-1TCF1-13) without cold acclimation. (B) Semi-quantitative RT-PCR for TCF1 expression in the wild type and transgenic plants. The three-week-old plants were treated with or without a seven-day cold acclimation at 4°C, respectively. Lane 1: WT, lane 2: tcf1-1, lane 3: tcf1-1Vector-2, lane 4: tcf1-1TCF1-3, lane 5: tcf1-1TCF1-12, lane 6: tcf1-1TCF1-13. (C) Quantification of survival rate of cold acclimated (black) and non-acclimated (grey) plants after freezing treatment at -8°C for 2 h and a seven day period of recovery. 1: WT, 2: tcf1-1, 3: tcf1-1Vector-2, 4: tcf1-1TCF1-3, 5: tcf1-1TCF1-12, 6: tcf1-1TCF1-13. Error bars are standard deviation (n = 80–100). Three biological experiments were performed and the data are expressed as mean ±S.E. Means with the same letter are not significantly different at P < 0.05 by One Way ANOVA analysis. (D) Leakage of electrolytes in WT, tcf1-1 and TCF1 complementary lines (tcf1-1TCF1-3, tcf1-1TCF1-12 and tcf1-1TCF1-13) treated at indicated temperatures below freezing. 3-week-old plants were cold-acclimated at 4°C for 7 day (Cold) or without treatment. Error bars are standard deviation (n = 10), (*, P < 0.05, t-test). (TIF) [file pgen.1005471.s004.tif]

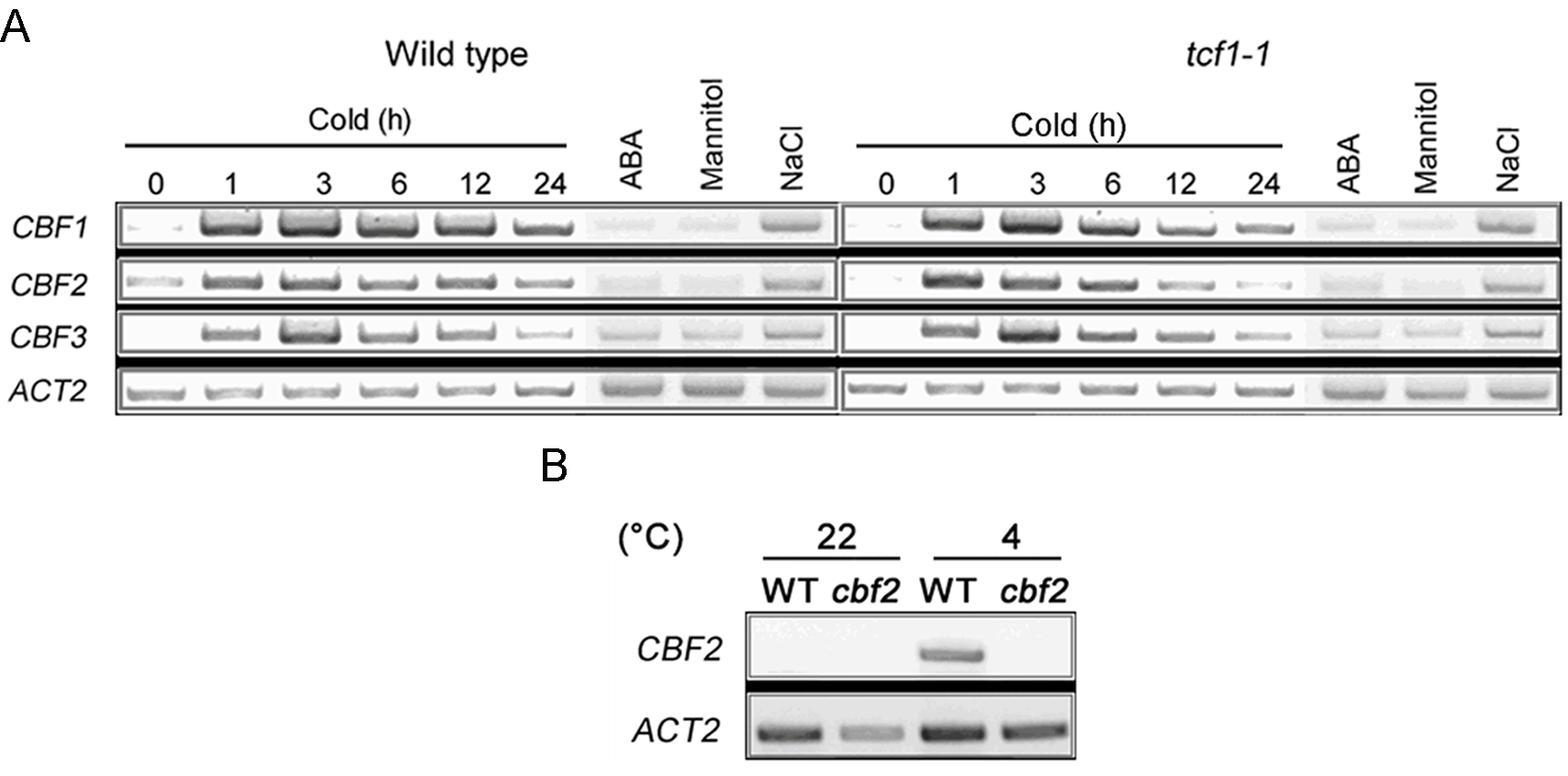

Supplement: S5 Fig — (A) The three week-old tcf1-1 and wild type plants were treated with cold at 4°C and rosettes were harvested at the indicated time points for RNA extraction and semi-quantitative RT-PCR. Rosettes of the plants treated with 100 μM ABA, 400 mM mannitol or 300 mM NaCl for 3 h were also collected for gene expression analysis. Transcript levels were assayed for CBF genes and loading control ACTIN2. (B) Semi-quantitative RT-PCR for CBF2 expression in a T-DNA insertion line. Three-week-old SALK_025203 and Col-0 seedlings grown on soil were subjected to low temperature (4°C) for 3 h, and the shoots were collected for the expression analysis. ACT2 gene was used as a loading control. The result showed that the mutant is a null mutation for the CBF2 gene, and was named as cbf2. (TIF) [file pgen.1005471.s005.tif]

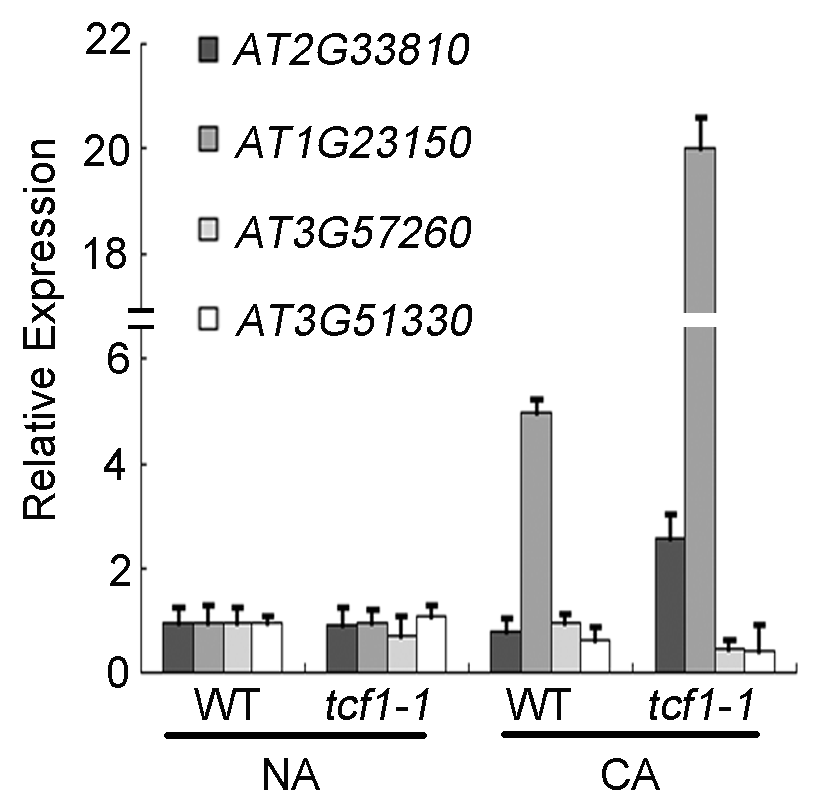

Supplement: S6 Fig — Three-week-old WT and tcf1-1 grown on MS medium plates were treated with cold stress for 7 day, and the levels of four genes’ transcripts were analyzed. The genes showed altered transcript levels in tcf1-1 in the microarray analysis were selected. ACT2 gene was used as a loading control. CA: Cold acclimation, NA: None cold acclimation. (TIF) [file pgen.1005471.s006.tif]

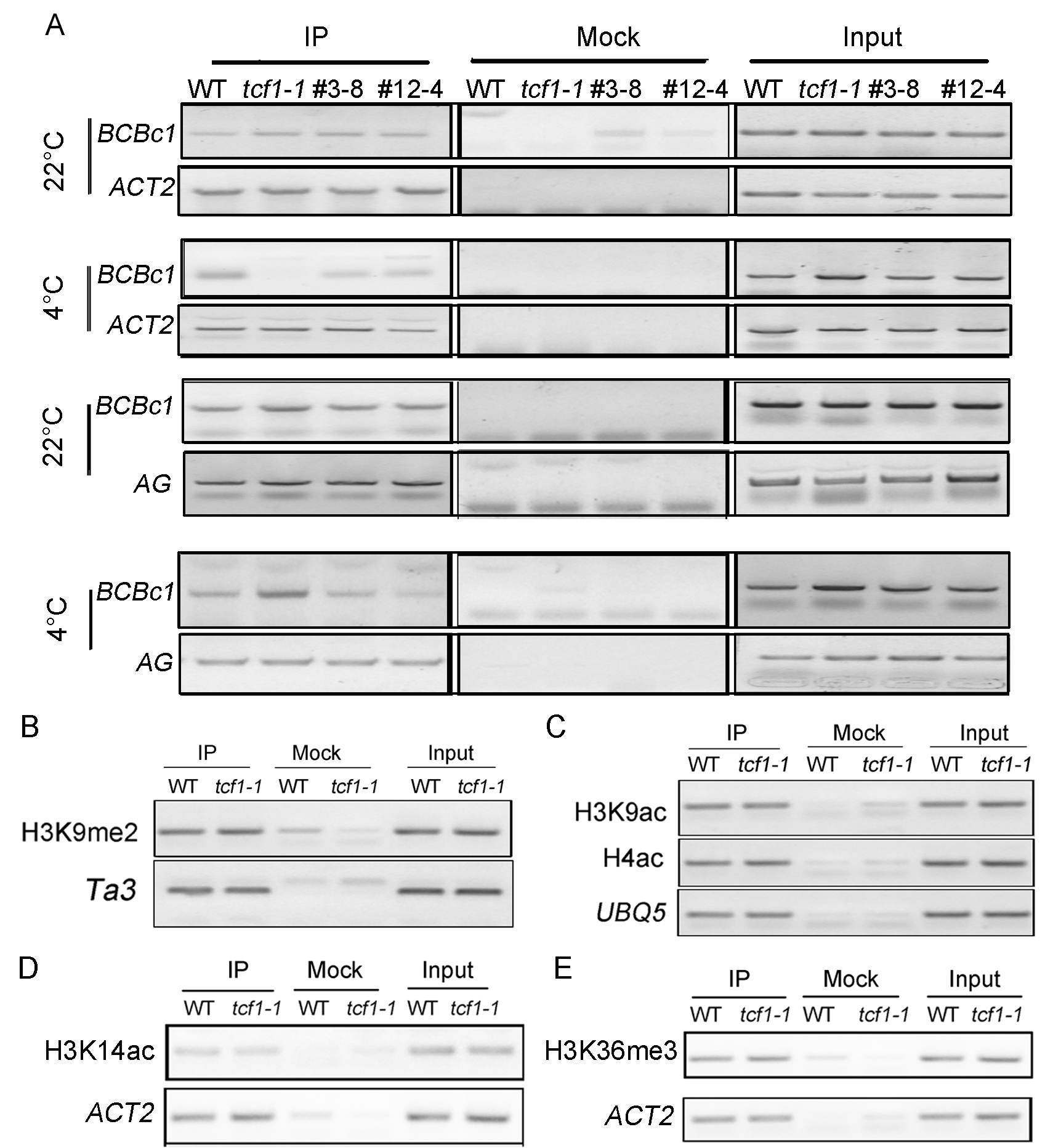

Supplement: S7 Fig — (A) Levels of H3K4me2 and H3K27me3 by ChIP-PCR analysis with wild-type (WT), tcf1-1, tcf1-1TCF1-3 and tcf1-1TCF1-12 plants normalized to ACT2 or AGAMOUS in BCBc1 fragment with and without a 7-day cold treatment. (B) to (E) Levels of H3K9me2, H3K9ac, H4Ac (The level of global histone H4 tetra-acetylation at K5/K8/K12/K16), H3K14ac and H3K36me3 by ChIP-PCR analysis normalized to indicated control in BCBc1 fragment in wild type and tcf1-1 plants with a seven-day cold acclimation. (TIF) [file pgen.1005471.s007.tif]

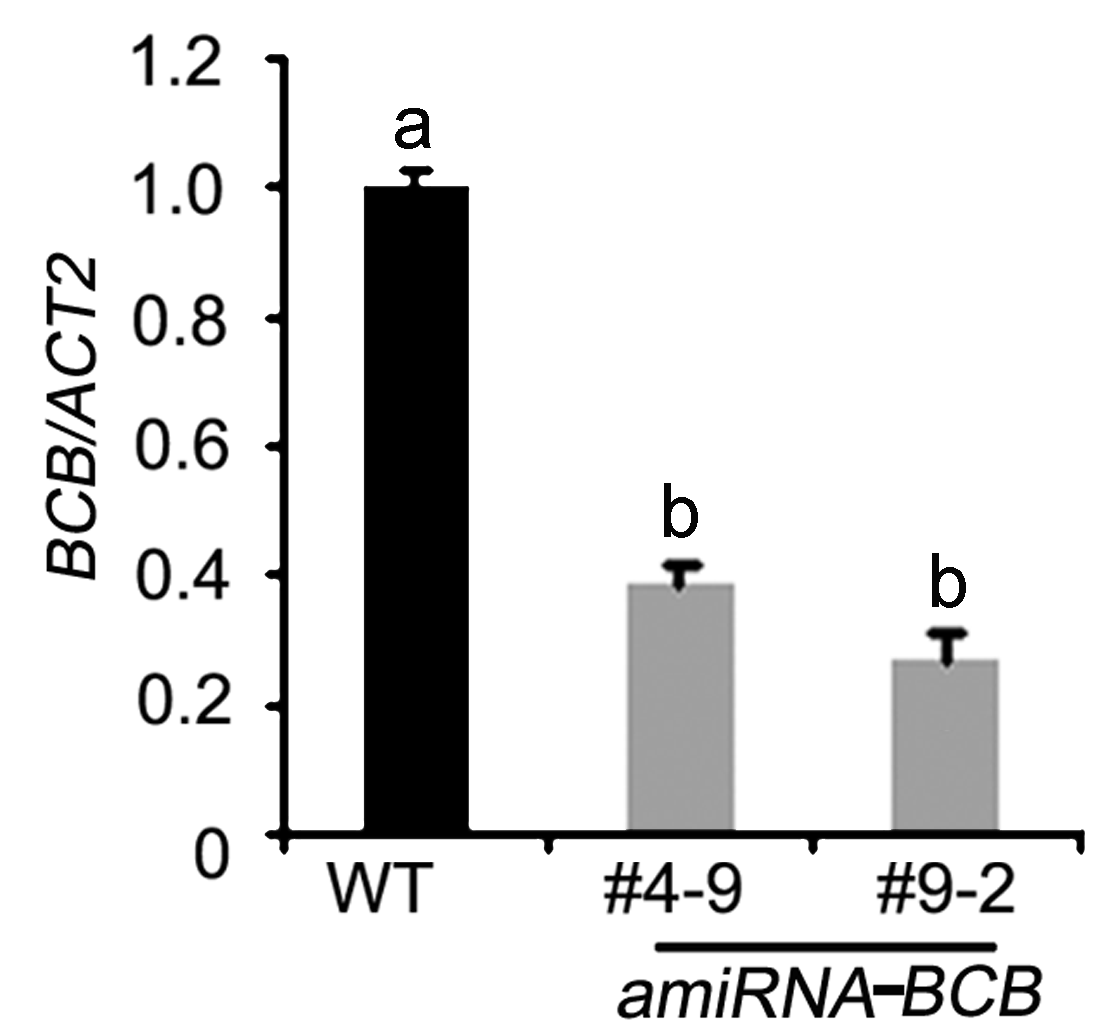

Supplement: S8 Fig — The levels of BCB transcripts in wild-type (Black) and amiRNA-BCB (Grey) plants. The plants were treated with cold at 4°C for 7 days. Three biological experiments were performed and the data are expressed as mean ±S.E. Means with the same letter are not significantly different at P < 0.05 by One Way ANOVA analysis. (TIF) [file pgen.1005471.s008.tif]

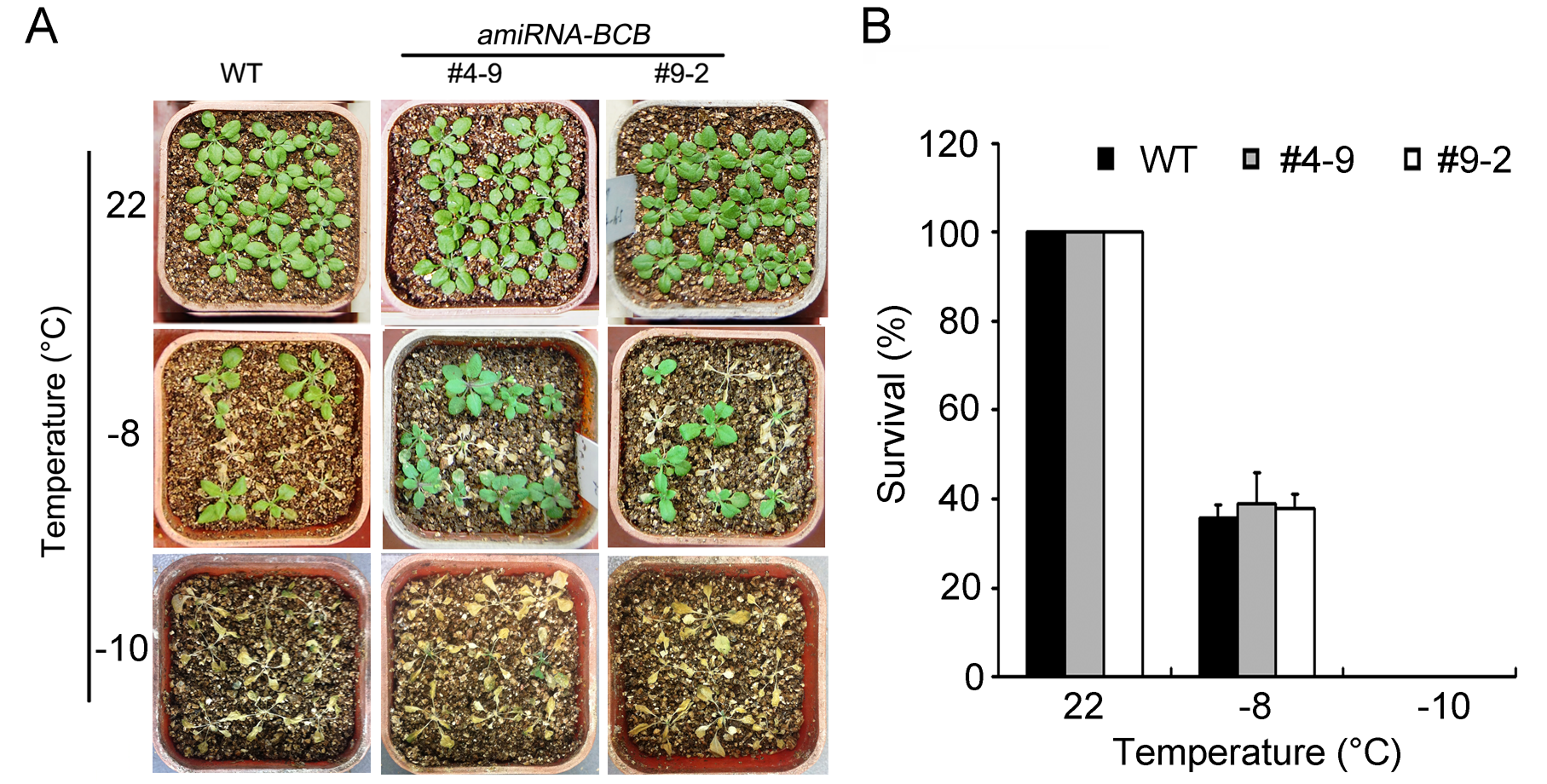

Supplement: S9 Fig — (A) Freezing treatment of three-week-old amiRNA-BCB transgenic plants (#4–9 and #9–2) and wild-type (WT) plants at the indicated temperature without cold acclimation. The pictures were taken 7-days after treatments. (B) Quantification of survival of the plants in (A). Error bars are standard deviation (n = 80–100). (TIF) [file pgen.1005471.s009.tif]

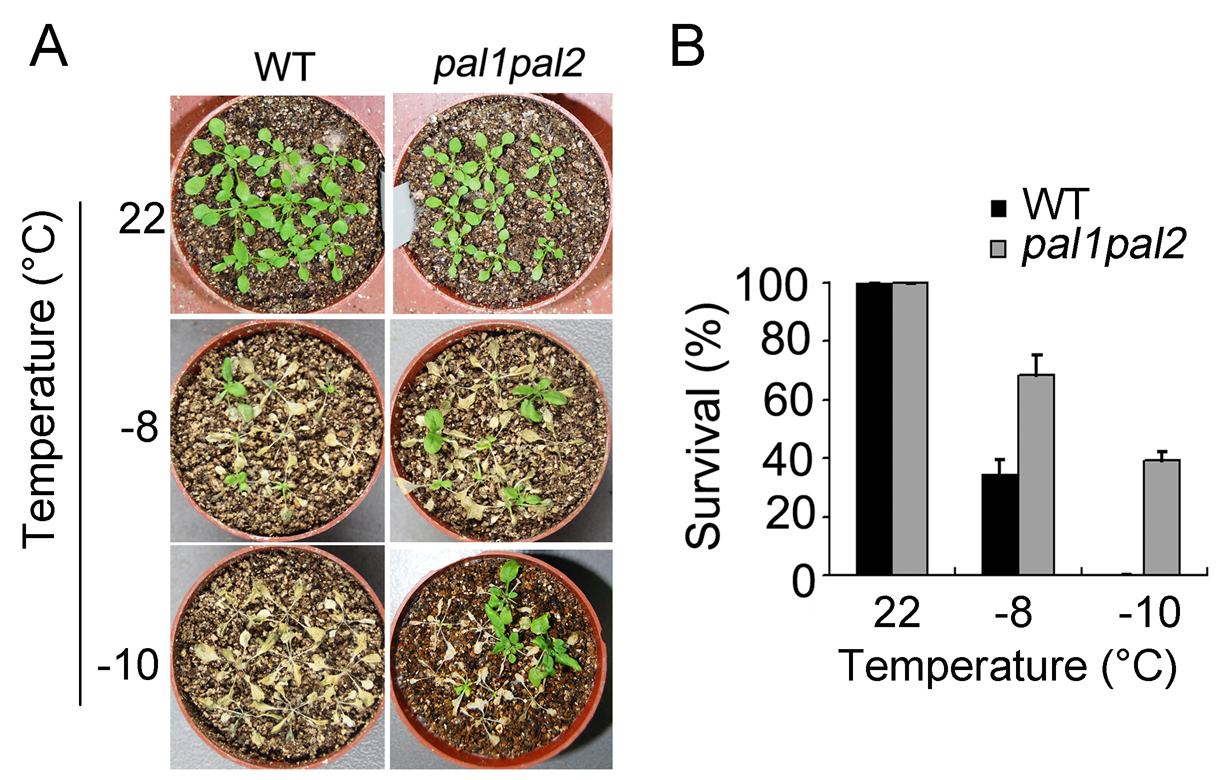

Supplement: S10 Fig — (A) Three week-old pal1pal2 and wild type (WT) plants without a 7-day cold treatment were used for freezing treatments at indicated time. The pictures were taken 7 days after treatments. (B) Quantification of survival of the wild type (Black) and pal1pal2 plants (Grey) in (A). Error bars represent standard deviation (n = 80–100). (TIF) [file pgen.1005471.s010.tif]
