# Supplementary material for: The Arabidopsis RCC1 Family Protein TCF1 Regulates Freezing Tolerance and Cold Acclimation through Modulating Lignin Biosynthesis
Source: PLoS Genet. 2015 Sep 22;11(9):e1005471. doi: 10.1371/journal.pgen.1005471 (PMC4579128; doi:10.1371/journal.pgen.1005471)
Supplement: S1 Table — (DOC) [file pgen.1005471.s011.doc]

**S1_Table**

Genes with increased and decreased expression levels by 1.5-fold in *tcf1-1* without cold acclimation determined by Biocapital Jingxin microarray

| **Gene ID** | **Score(d)** | **Description** |
| --- | --- | --- |
| **Genes with increased expression levels by 1.5-fold in *tcf1-1*** | | |
| At1g47980 | 2.880625606 | similar to unknown protein(AT3G62730.1) |
| At2g07739 | 2.516311376 | similar to unknown protein (AT2G07739.1) |
| At2g26695 | 2.057341084 | zinc finger (Ran-binding) family protein |
| At5g58320 | 1.993613527 | ATEM6 (Arabidopsis Early Methionine-Labelled 6) |
| At3g11980 | 1.859096285 | MS2 (Male Sterility 2) |
| At5g53450 | 1.826873629 | ORG1 (OBP3-Responsive Gene 1) |
| At5g13740 | 1.715241612 | ZIF1 (Zinc Induced Facilitator 1) |
| At5g05250 | 1.582234197 | similar to unknown protein (AT3G56360.1) |
| **Genes with decreased expression levels by 1.5-fold in *tcf1-1*** | | |
| At2g19800 | -2.388083481 | MIOX2 (Myo-Inositol Oxygenase 2) |
| At3g25050 | -2.134781598 | XTH3 (Xyloglucan Endotransglucosylase/Hydrolase 3) |
| At2g25610 | -2.096670168 | H+-transporting two-sector ATPase, C subunit family protein |
| At1g08830 | -1.99126327 | CSD1 (copper/zinc superoxide dismutase 1); copper, zinc superoxide dismutase |
| At5g41010 | -1.91038969 | DNA-directed RNA polymerases I, II, and III 7 kDa subunit, putative |
| At1g75910 | -1.737865826 | EXL4 (extracellular lipase 4); acyltransferase/ carboxylic ester hydrolase/ lipase |
| At5g19770 | -1.715801905 | TUA3 (tubulin alpha-3) |
| At5g50900 | -1.654898811 | armadillo/beta-catenin repeat family protein |
| At1g32410 | -1.648652706 | vacuolar protein sorting 55 family protein / VPS55 family protein |
| At3g57260 | -1.624804279 | BGL2 (Pathogenesis-Related Protein 2) |
| At3g50830 | -1.623219103 | COR413-PM2 (cold regulated 413 plasma membrane 2) |
| At5g10760 | -1.535288527 | aspartyl protease family protein |
