# Supplementary material for: The Arabidopsis RCC1 Family Protein TCF1 Regulates Freezing Tolerance and Cold Acclimation through Modulating Lignin Biosynthesis
Source: PLoS Genet. 2015 Sep 22;11(9):e1005471. doi: 10.1371/journal.pgen.1005471 (PMC4579128; doi:10.1371/journal.pgen.1005471)
Supplement: S2 Table — (DOC) [file pgen.1005471.s012.doc]

**S2_Table**

Genes with increased and decreased expression levels by 1.5-fold in *tcf1-1* with cold acclimation determined by Biocapital Jingxin microarray

| **Gene ID** | **Scord(d)** | **Description** |
| --- | --- | --- |
| **Genes with increased expression levels by 1.5-fold in *tcf1-1*** | | |
| At1g23150.1 | 7.150168726 | similar to putative senescence-associated protein |
| At2g33810.1 | 3.017897019 | SPL3 (Squamosa Promoter Binding Protein-Like 3) |
| At1g69120.1 | 2.296107305 | AP1 (APETALA1) |
| At1g53160.1 | 1.830154766 | SPL4 (Squamosa Promoter Binding Protein-Like 4) |
| At1g48750.1 | 1.722067297 | protease inhibitor/seed storage/lipid transfer protein (LTP) family protein |
| At1g47980.1 | 1.64581716 | similar to H0502B11.4 (Oryza sativa,GB:CAH66524.1) |
| At5g50720.1 | 1.635279444 | ATHVA22E (Arabidopsis thaliana HVA22 homologue E) |
| At3g13060.1 | 1.617802892 | ECT5 |
| At3g19030.1 | 1.608936143 | similar to unknown protein(AT1G49500) |
| At1g72430.1 | 1.567723993 | auxin-responsive protein-related |
| At1g07820.1 | 1.55731865 | histone H4 |
| At5g60400.1 | 1.543148134 | similar to hypothetical protein (Medicago truncatula,GB:ABE89561.1) |
| At5g59690.1 | 1.518311863 | histone H4 |
| At5g64770.1 | 1.517348717 | similar to 80C09_10 (Brassica rapa subsp. Pekinensis,GB:AAZ41821.1) |
| **Genes with decreased expression levels by 1.5-fold in *tcf1-1*** | | |
| At3g57260.1 | -2.451982366 | BGL2 (Pathogenesis-Related Protein 2) |
| At3g51330.1 | -2.391836776 | aspartyl protease family protein |
| At1g30730.1 | -2.333626116 | FAD-binding domain-containing protein |
| At4g11890.1 | -2.137870696 | protein kinase family protein |
| At5g16970.1 | -2.110507287 | AT-AER (ALKENAL REDUCTASE); 2-alkenal reductase |
| At1g78830.1 | -2.026731464 | curculin-like (mannose-binding) lectin family protein |
| At5g10760.1 | -2.019519713 | aspartyl protease family protein |
| At5g24210.1 | -1.834506752 | lipase class 3 family protein |
| At1g75040.1 | -1.833701554 | PR5 (Pathogenesis-Related Gene 5) |
| At2g18690.1 | -1.788868261 | similar to unknown protein(AT2G18680.1) |
| At2g43570.1 | -1.75880001 | chitinase, putative |
| At4g20830.2 | -1.751841749 | FAD-binding domain-containing protein |
| At5g06630.1 | -1.742689716 | proline-rich extensin-like family protein |
| At3g13790.1 | -1.722083477 | ATBFRUCT1/ATCWINV1 (Arabidopsis Thaliana Cell Wall Invertase 1) |
| At2g14560.2 | -1.691930038 | similar to unknown protein (AT1G33840.1) |
| At1g44750.1 | -1.66179657 | ATPUP11 (Arabidopsis thaliana purine permease 11) |
| At2g17040.1 | -1.637277888 | ANAC036 (Arabidopsis NAC domain containing protein 36) |
| At1g70520.1 | -1.614370355 | protein kinase family protein |
| At3g14620.1 | -1.608960002 | CYP72A8 (cytochrome P450, family 72, subfamily A, polypeptide 8) |
| At1g30720.1 | -1.602629223 | FAD-binding domain-containing protein |
| At3g01290.1 | -1.567758612 | band 7 family protein |
| At5g20230.1 | -1.559669414 | ATBCB (Arabidopsis Blue-Copper-Binding Protein); copper ion binding |
| At2g22500.1 | -1.503101851 | mitochondrial substrate carrier family protein |
