# Supplementary material for: The Arabidopsis RCC1 Family Protein TCF1 Regulates Freezing Tolerance and Cold Acclimation through Modulating Lignin Biosynthesis
Source: PLoS Genet. 2015 Sep 22;11(9):e1005471. doi: 10.1371/journal.pgen.1005471 (PMC4579128; doi:10.1371/journal.pgen.1005471)
Supplement: S3 Table — (DOC) [file pgen.1005471.s013.doc]

**S3_Table** List of Primers

|  |  |  |  |
| --- | --- | --- | --- |
| **Name** | **Sequence** | **Gene** | **Purpose** |
| At4g25490 QF | CTTCGCTGACTCGGCTTG | CBF1 | Real Time Q-PCR |
| At4g25490 QR | CCACCATCGTCTCCTCCA |  |  |
| At4g25470 QF | GAGGATTTGGCTCGGGAC | CBF2 | Real Time Q-PCR |
| At4g25470 QR | TGGCACAGGTTGATTCCG |  |  |
| At4g25480 QF | TGAGATGTGTGATGCGACG | CBF3 | Real Time Q-PCR |
| At4g25480 QR | TTAGCCAACAAACTCGGCA |  |  |
| At5g52310 QF | GAAGATGATGATGATGACGAGC | RD29A | Real Time Q-PCR |
| At5g52310 QR | TCAGTGGGTTTGGTGTAATCG |  |  |
| At1g20440 QF | GAGCGATGAAGAAGGTGAGG | COR47 | Real Time Q-PCR |
| At1g20440 QR | CGGGATGGTAGTGGAAACTG |  |  |
| At2g42540 QF | CGCTAAAGGTGACGGCAA | COR15A | Real Time Q-PCR |
| At2g42540 QR | CCCAATGTATCTGCGGTTTC |  |  |
| At5g20230 QF | CGGAATGGACGAGACCTATG | BCB | Real Time Q-PCR |
| At5g20230 QR | GCTTCTGATACAACTGCCACAT |  |  |
| At2g37040 QF | TGGAGAGTATGAACAAAGGCAC | PAL1 | Real Time Q-PCR |
| At2g37040 QR | AGTGTGGCAATGTGTGGCT |  |  |
| At3g53260 QF | GGCACCGCATTACAAACAG | PAL2 | Real Time Q-PCR |
| At3g53260 QR | CGGAGTATCCTTGGAGAAGAGT |  |  |
| At5g04230 QF | TCGTCAACCAAACGCAACAG | PAL3 | Real Time Q-PCR |
| At5g04230 QR | AGTCACTACTCGCCTTCACAC |  |  |
| At3g10340 QF | CCAAAGCCGACAACAAGAGC | PAL4 | Real Time Q-PCR |
| At3g10340 QR | AGAGAGAGGAACAAGGTCACC |  |  |
| At1g80820 QF | AGGAGCCAAGGAAAGACTCAC | CCR2 | Real Time Q-PCR |
| At1g80820 QR | ATCGGAGAGGCAGTGTGGAA |  |  |
| At4g25490 F | TCCGATTACGAGCCTCAAGG | CBF1 | RT-PCR |
| At4g25490 R | CGGCATCCCAAACATTGTCT |  |  |
| At4g25470 F | AACCAGCGGGAAGGAAGAAG | CBF2 | RT-PCR |
| At4g25470 R | AAATAGCCTCCACCAAGGTCT |  |  |
| At4g25480 F | CTGAAATGTTTGGCTCCG | CBF3 | RT-PCR |
| At4g25480 R | TTCCGCCGTGTAAATAGC |  |  |
| At2g42540 F | GCTGTTCTCACTGGTATGGC | COR15A | RT-PCR |
| At2g42540 R | TTGTGGCATCCTTAGCCTCTC |  |  |
| At2g42530 F | TGGGTTCTTCTTTCCACAACG | COR15B | RT-PCR |
| At2g42530 R | GGCATTCTTAGCCTCTTCTGC |  |  |
| At1g20440 F | GTCGCAACAGAGGAATCACC | COR47 | RT-PCR |
| At1g20440 R | GTTACAACCAACGGCGTGGA |  |  |
| At1g20450 F | GCGTCTGAGTTTGAGCACAA | ERD10 | RT-PCR |
| At1g20450 R | CCAGTGGTCTTGGCGTGATA |  |  |
| At5g52310 F | GGTGTTTCCTGTCGTGTCT | RD29A | RT-PCR |
| At5g52310 R | TTCTCCGTCAAATCCCGTC |  |  |
| At3g55580 F | ATGAATGGCGAGGGAAAATTG | TCF1 | RT-PCR |
| At3g55580 R | TCA1TAGAGTTGGTTGGCC |  |  |
| At5g54640 F | GGAATTCCATATGATGGCTGGTCGTGGAAAAAC | HTA1 | Yeast two Hybrid |
| At5g54640 R | CCGCTCGAGCTAATCTTCCTGAGGCTTTG |  |  |
| At5g10400 F | GGAATTCCATATGATGGCTCGTACCAAGCAAAC | HTR9 | Yeast two Hybrid |
| At5g10400 R | CCGCTCGAGTTAAGCCCTCTCGCCTCTAATTC |  |  |
| At5g59690 F | GAATTCA1TGTCGGGTCGTGGAAAGGG | HFO2 | Yeast two Hybrid |
| At5g59690 R | GTCGACTTAACCACCGAATCCGTAAAG |  |  |
| At1g07820 F | GAATTCA1TGTCGGGAAGAGGAAAGGG | HFO4 | Yeast two Hybrid |
| At1g07820 R | GTCGACTTAACCTCCGAAACCGTAGAG |  |  |
| At3g55580GUS F | AAGCTTTTTGCCACCTGTCTGTTTAG | TCF1 | *ProTCF1::GUS construct* |
| At3g55580GUS R | GGATCCTTTTCTCAGTTTTGAAAACCTGACG |  |  |
| At3g55580GST F | GCGGATCCATGAATGGCGAGGGAAAATTG |  | GST-TCF1 construct |
| At3g55580GST R | GCGTCGACTCATAGAGTTGGTTGGCC |  |  |
| At3g55580Comp F | GCGGATCCATGAATGGCGAGGGAAAATTG |  | gene complementation |
| At3g55580Comp R | GCGTCGACTCATAGAGTTGGTTGGCC |  |  |
| At3g55580GFP F | GCGAATTCATGAATGGCGAGGGAAAATTG |  | GFP-TCF1 construct |
| At3g55580GFP R | GCGGATCCTCATAGAGTTGGTTGGCC |  |  |
| At3g55580NativePro F | GAGCTCTTTGCCACCTGTCTGTTTAG |  | CHIP assay |
| At3g55580NativePro R | AAGCTTTTTTCTCAGTTTTGAAAACCTGACG |  |  |
| At5g20230 CF1 | TTATGACCGCATTCGTTTAC | BCBp1 | CHIP assay |
| At5g20230 CR1 | AGCGCGTAAAGACATTGTAC |  |  |
| At5g20230 CF2 | GATACGGAATGGACGAGAC | BCBc1 |  |
| At5g20230 CR2 | AACATCAGTCGGTGGAAAC |  |  |
| At5g20230 CF3 | ATCACTGTAGTTGCTGCTGGT | BCBc2 |  |
| At5g20230 CR3 | TGAAGGTGTTGTGGTCCCA |  |  |
